# Using sensitivity analysis to identify key factors for the propagation of a plant epidemic

**DOI:** 10.1098/rsos.171435

**Published:** 2018-01-17

**Authors:** Loup Rimbaud, Claude Bruchou, Sylvie Dallot, David R. J. Pleydell, Emmanuel Jacquot, Samuel Soubeyrand, Gaël Thébaud

**Affiliations:** 1 BGPI, INRA, Montpellier SupAgro, University of Montpellier, CIRAD, TA A-54/K, Campus de Baillarguet, Montpellier Cedex 5, 34398, France; 2 BioSP, INRA, Avignon, 84914, France

**Keywords:** heterogeneous landscape, polynomial regression, sensitivity index, Sobol's method, simulation model, spatially explicit model

## Abstract

Identifying the key factors underlying the spread of a disease is an essential but challenging prerequisite to design management strategies. To tackle this issue, we propose an approach based on sensitivity analyses of a spatiotemporal stochastic model simulating the spread of a plant epidemic. This work is motivated by the spread of sharka, caused by *plum pox virus*, in a real landscape. We first carried out a broad-range sensitivity analysis, ignoring any prior information on six epidemiological parameters, to assess their intrinsic influence on model behaviour. A second analysis benefited from the available knowledge on sharka epidemiology and was thus restricted to more realistic values. The broad-range analysis revealed that the mean duration of the latent period is the most influential parameter of the model, whereas the sharka-specific analysis uncovered the strong impact of the connectivity of the first infected orchard. In addition to demonstrating the interest of sensitivity analyses for a stochastic model, this study highlights the impact of variation ranges of target parameters on the outcome of a sensitivity analysis. With regard to sharka management, our results suggest that sharka surveillance may benefit from paying closer attention to highly connected patches whose infection could trigger serious epidemics.

## 1. Introduction

Many factors impact the spread of a plant disease. Such factors include the biology of the different species involved in the pathosystem, the processes governing primary and secondary infections and, generally, landscape structure and composition [1]. Precisely identifying the key factors for disease spread is of prime interest for disease management, because they constitute a target for research efforts and control strategies [2,3]. However, the identification of the key factors of an epidemic through experiments rapidly becomes intractable, because there can be a huge number of candidate parameters, not counting their interactions. In addition, epidemics must be considered at a large spatiotemporal scale, which is rarely possible in experiments (but see e.g. [4]). Expert opinions are often simple, efficient and quick alternatives, but are by their nature prone to error. Finally, epidemiological models are an interesting approach because of their ability to test several scenarios, while circumventing the difficulties associated with experiments.

Global sensitivity analysis of a simulation model aims to assess the fraction of the variability of the output that can be attributed to each input parameter, and thus to rank these parameters by influence on the model output [2,5,6]. This method is based on a numerical experiment designed to explore the parameter space using numerous parameter combinations. In ecological modelling, possible values of each input parameter traditionally come from the literature or via expert knowledge, and are often summarized by bounded intervals [6]. Global sensitivity analysis has been very useful in identifying key factors behind the spread of different animal pathogens [7,8] and their vectors [9], as well as of invasive plants [10]. In plant epidemiology, some studies dealing with plant viruses [11–13] and fungi [14,15] used formal sensitivity analyses; however, the developed approaches did not allow the inclusion of all candidate epidemiological processes and interactions.

A panel of statistical methods has been proposed for global sensitivity analysis of deterministic models during the last 30 years. Available approaches mainly include graphical methods to explore marginal effects, the elementary effects method [16], variance-based methods or metamodelling, i.e. fitting of a mathematical function which approximates the model [5,17,18]. By contrast, few methods have been proposed for stochastic models [7,8,15], despite their wide use in ecology and epidemiology. Model stochasticity accounts for variability and uncertainty in the different biological processes [1]. Stochastic models are thus essential when performing epidemiological risk assessment.

The first aim of this study was to present a rigorous framework for the sensitivity analysis of a stochastic spatially explicit model simulating the spread of a plant disease, and to better understand the influence of different epidemiological parameters on model outputs. The second objective was to uncover the key parameters of epidemic spread. As a case study, this work focused on sharka, caused by *plum pox virus* (PPV, genus *Potyvirus*, family *Potyviridae*), one of the most damaging diseases affecting trees of the genus *Prunus* (e.g. peach, apricot and plum) [19,20]. Depending on virus–host (or virus–cultivar) interactions, fruits of symptomatic hosts can be unmarketable due to considerable alterations or premature drop [21]. The disease has dispersed worldwide owing to commercial exchanges of contaminated plant material [20]. In addition, PPV is naturally transmitted from infectious sources to healthy trees by many aphid species in a non-persistent manner [20,22,23]. According to this transmission mechanism, an aphid vector can acquire viral particles from an infected host and may inoculate the virus to a susceptible host for a short period of time [24]. Once inoculated—and after a latent period—new infected hosts constitute in turn viral sources for aphid-mediated transmission. For PPV, it is commonly assumed, and was recently shown with young peach plants, that the beginning of the infectious period occurs at the same time as symptom expression (i.e. the end of the incubation period) on the infected host [25,26]. Prevalence at introduction in orchards can be extremely variable depending on the type of contamination in nurseries. If only some plants intended for planting are contaminated by infectious aphids, few infected trees will be planted in orchards. On the contrary, if a tree used as propagation stock is infected, the whole batch of progeny plants intended for planting will be infected, resulting in a massive introduction at orchard planting.

An explicit spatiotemporal simulation model was previously developed and used to estimate biological parameters of sharka spread in a real landscape using data from PPV (M strain) epidemics in a French peach-growing area [27]. It is based on an SEIR architecture, i.e. each host can be classified in one of the following states: *S* (susceptible: healthy), *E* (exposed: infected but not yet infectious nor symptomatic), *I* (infectious and symptomatic), *R* (removed). Transitions between states are defined by stochastic equations. Through Markov chain Monte Carlo simulations within a Bayesian framework, this model enabled estimating the transmission coefficient (i.e. the transmission intensity per infectious tree), the duration of the latent period of PPV infection, and the dispersal kernel of infectious aphids [27]. In a landscape (i.e. in two dimensions), a dispersal kernel can be defined as the probability distribution of the position of a particle (e.g. an insect) after dispersal from the origin [28].

In the present study, this simulation-based estimation model was modified in order to perform two different sensitivity analyses, destined to highlight the impact of the variation ranges of the target parameters. Global sensitivity analyses generally only focus on a specific system and use narrow variation ranges for well-known parameters and wider variation ranges to encompass all possible values of less-known parameters (e.g. [29,30]). In contrast, here we intended to treat equally all target parameters by using wide and narrow variation ranges in two separate analyses. Thus, the first analysis (called ‘broad-range sensitivity analysis’ hereafter) was designed to assess the intrinsic influence of different epidemiological parameters on the overall behaviour of the model, ignoring any prior information on parameter values. The range of variation for each target parameter was very wide and covered, when possible, the whole definition domain. The second, sharka-specific, analysis benefited from available knowledge on the epidemiological parameters of PPV in southeastern France (published data and expert opinions were jointly used to obtain biologically reasonable bounds for the variation ranges of each parameter). On the one hand, the first analysis demonstrates the interest of sensitivity analyses based on Sobol's indices and metamodelling to explore the properties of a stochastic model. The second, sharka-specific, sensitivity analysis uncovers the most influential epidemiological parameters, which may require further knowledge from research work, or further action for sharka management in peach orchards.

## 2. Model description

The model is stochastic, orchard-based, with a discrete time step of 1 week denoted *t* (*t* = 1, … , *T*). Simulations run for a period of 35 years (from *a*_0_ = 1 to *a*_f_ = 35), which is a reasonable duration to assess the long-term impact of an epidemic in cultivated perennial plants.

### 2.1. Landscape and orchard dynamics

Orchard data were collected in a peach-growing area in southeastern France. The resulting database includes 34 years of information on 553 patches (i.e. pieces of land); the distance between the centroids of neighbouring patches ranges from 16 to 496 m (median: 55 m). On these patches, 597 peach orchards (i.e. homogeneous crop of a single cultivar with the same planting date) have been continuously cultivated (i.e. removed orchards are replanted during the next winter). In our model, we use the real landscape of 553 patches (electronic supplementary material, figure S1) which has been used previously to estimate most parameters of the model [27]. However, we simulate new orchard dynamics on the patches. To stabilize the mean age of the orchards at year *a*_0_ = 1, an initial orchard planting year is uniformly drawn between −39 and −20 for each patch. Then the following algorithm is iterated: (i) the orchard duration (after which the orchard is removed) is drawn from a Poisson distribution whose mean is *ψ* = 15 years; (ii) the year after orchard removal gives the planting year of the subsequent orchard on the same patch. For each orchard, a planting density is randomly drawn from the empirical distribution of the planting densities of the 597 orchards in the database.

### 2.2. SEI architecture

**Figure 1.**
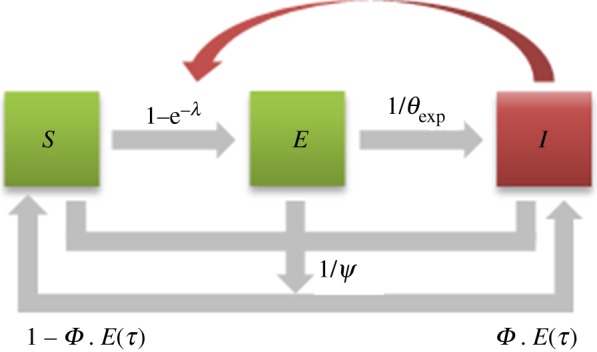
Representation of the spatiotemporal stochastic simulation model as a flow diagram. *S*, susceptible (i.e. healthy); *E*, exposed (i.e. infected but neither infectious nor diseased); *I*, infectious and symptomatic. Productive and non-productive (diseased) hosts are in green and red, respectively. Infection of healthy hosts depends on the infectious potential (*λ*) applied by infectious hosts and their spatial location. Infected hosts become infectious after a latent period (with a mean rate 1/*θ*_exp_). Regardless of their sanitary status, hosts can be removed due to orchard turnover (with a mean rate *1/ψ*). Each orchard planting has a mean risk of introduction (*ϕ*) of infectious trees (whose mean proportion in the orchard is *E*(*τ*)).

Each host is in one of the following three different health states (figure 1): *S* (susceptible), *E* (exposed) or *I* (infectious). In orchard *i* at time step *t*, let the variables *S_i,t_*, *E_i,t_* and *I_i,t_* describe the respective number of hosts in each state (*S_i,t_* + *E_i,t_* + *I_i,t_* is the total number of living hosts). Based on the characteristics of non-persistent transmission, a viruliferous vector is considered non-infectious once it has probed (and may have transmitted the pathogen) on a first healthy host. Thus, the number of hosts being infected (i.e. moving from *S* to *E*) between *t* and *t* + 1 in orchard *i* is defined as follows:

$$[S_{i,t}\to E_{i,t+1}]∼\text{Binomial(}S_{i,t},1-{\text{e}}^{-\lambda_{i,t}}),$$

with *λ_i,t_* the infectious potential incurred by each tree of the orchard due to the presence of infectious hosts inside the orchard as well as in other source orchards. This infectious potential is calculated as:

$$\lambda_{i,t}=\frac{\beta .\alpha_{t}}{S_{i,t}+E_{i,t}+I_{i,t}}.\sum_{i^{\prime }}m_{i^{\prime }i}.I_{i^{\prime },t},$$

where *β* is the transmission coefficient (i.e. the number of vectors leaving a given infectious host over one year and able to initiate an infection on a healthy host); *α_t_* is the normalized flight density (i.e. the proportion of annual flights that occur at time step *t*); and *m_i′i_* is the connectivity between orchards *i′* and *i*. This connectivity gives the probability that a vector disperses from orchard *i′* to orchard *i*, and depends on the dispersal kernel of the vector. The division by *S_i,t_* + *E_i,t_* + *I_i,t_* allows the expression of *λ_i,t_* as a mean infectious potential per host in the recipient orchard.

Infected hosts become infectious (i.e. move from state *E* to state *I*) after a latent period whose duration is given by $(EI)=\text{max(}A_{1},A_{2}\text{)}$ with $A_{1}\;∼\;\text{Gamma(}\theta_{1},\theta_{2}\text{)}$ and *A*_2_ the duration between the inoculation date and the end of the year. The minimal delay imposed by the term *A*_2_ was introduced because, in natural conditions, the latent period of sharka does not end within the growing season when the tree is inoculated [21,31,32].

It is assumed that trees in state *I* are not productive any more, due either to the absence of yield induced by the disease, or to the ban on fruit sales from symptomatic trees. However, the disease is not supposed to affect host lifespan (no available data reported any increase in peach mortality due to PPV), and this work is focused on disease spread in the absence of any control action (e.g. removal of diseased trees). Consequently, this model does not include an *R* (removed) state. Nevertheless, as defined by the algorithm used to simulate orchard turnover (see ‘Landscape and orchard dynamics’), orchards are regularly renewed (due to agricultural reasons, regardless of the disease) with trees in state *S* (or possibly *I*, see ‘Pathogen introduction’) (figure 1).

### 2.3. Pathogen dispersal

The calculation of the connectivity matrix, *m*, requires the integration of the dispersal kernel, *f*, over all the points *p*_1_ in orchard *i* (whose patch is represented by polygon *A_i_*) and the points *p*_2_ in orchard *i′* (whose patch is represented by polygon *A_i′_*):

$$m_{i^{\prime }i}=\frac{\int_{p_{2}\in A_{i^{\prime }}}\int_{p_{1}\in A_{i}}f(||p_{2}-p_{1}||).\text{d}p_{1}.\text{d}p_{2}}{\int_{p_{2}\in A_{i^{\prime }}}\text{d}p_{2}},$$

with $||p_{2}-p_{1}||=d$ the Euclidean distance between points *p*_1_ and *p*_2_. For a given dispersal kernel, this calculation is performed using the CaliFloPP algorithm [33]. However, its computational cost is huge, and consequently not affordable if the dispersal kernel varies among the different simulations of a sensitivity analysis. Therefore, the dispersal kernel, which is isotropic in this work (i.e. we assume that aphid dispersal is homogeneous in all directions), is defined as a weighted mixture of *J* exponential distributions using the same approach as in [27]:

$$f(d)=\sum_{j=1}^{J}w_{j}.\frac{{\text{e}}^{-(d/h_{j})}}{2.\pi .h_{j}^{2}},$$

with $h_{j}=(3\text{/}2)\times 1.47^{j-1}$ the distance parameter and $w_{j}=\int_{(j-1)/J}^{j/J}g(x;\;s_{1},s_{2}).\text{d}x$ the weight of each exponential distribution. Using *J* = 20, the equation governing *h_j_* enables the use of exponential distributions whose means increase exponentially from 3 m to 4.5 km, i.e. from approximately the mean distance between two trees in an orchard to the dimension of the simulated landscape. To manipulate the weights of these *J* exponential distributions via just two parameters (*s*_1_ and *s*_2_), and to guarantee that they sum to 1, they are calculated by integrating the probability density *g*(*x; s*_1_, *s*_2_) of the variable $W∼\text{Beta}\;(s_{1};\;s_{2})$, hereafter called ‘dispersal weighting variable’, on *J* sub-intervals consisting in a partition of the interval [0; 1] (noting that the probability density of a beta distribution integrates to 1 on the interval [0; 1], see also electronic supplementary material, figure S2). The diversity of possible shapes of the beta distribution allows a lot of flexibility to define the different weights (see electronic supplementary material, figure S3C). Thus, the connectivity matrix used in the simulations is a weighted mixture of *J* = 20 matrices calculated beforehand with their respective exponential functions. The mean dispersal distance is: $\sum_{j=1}^{J}w_{j}.2.h_{j}$.

### 2.4. Pathogen introduction

At *t* = 1, the pathogen is introduced for the first time at orchard planting. In addition, starting from *t* = 1, every orchard planting (whatever the patch) is subjected to a risk *Φ* of new introduction. For each introduction, the probability that a given host is infected at orchard planting, *τ*, is described by a weighted mixture of two beta distributions:

$$\tau =(1-p_{\mathrm{MI}}).\;\tau_{1}+p_{\mathrm{MI}}.\tau_{2},$$

with $\tau_{1}∼\text{Beta}\;(\xi_{11};\;\xi_{12})$ and $\tau_{2}∼\text{Beta}\;(\xi_{21};\;\xi_{22})$. These two distributions represent two different patterns of pathogen introduction from contaminated nurseries, depending on whether the contamination occurred on plants for planting (leading to a weak prevalence at introduction) or on propagation stock (leading to a high prevalence). Thus *p*_MI_ indicates the relative probability of massive introduction. Infected hosts at planting are placed directly in state *I*. This assumption is more parsimonious than those needed to simulate the latent period duration of these trees, whose inoculation date (in nurseries) and process (from viruliferous aphids or from infected propagation stock) are unknown. The mean number of infectious trees at planting (in case of introduction) is: $E(\tau )=(1-p_{\mathrm{MI}}).\;(\xi_{11}\text{/}(\xi_{11}+\xi_{12}))+p_{\mathrm{MI}}.(\xi_{21}\text{/}(\xi_{21}+\xi_{22}))$. Parametrization of *τ*_1_ and *τ*_2_ is such that $\left(\xi_{21}\text{/}(\xi_{21}+\xi_{22}))>(\xi_{11}\text{/}(\xi_{11}+\xi_{12})\right)$.

To control the connectivity of the patch where the first introduction occurs, this patch is selected based on its theoretical ‘outgoing connectivity’. For each patch *i′*, this parameter, denoted *κ_i′_*, describes the mean number of infectious aphids that would leave patch *i′* if all trees in this patch were infectious, and land in any other patch of the landscape:

$$\kappa_{i^{\prime }}=\sum_{i\neq i^{\prime }}(m_{i^{\prime }i}\times N_{i^{\prime }}^{th})=A_{i^{\prime }}\times \mu^{th}\times \sum_{i\neq i^{\prime }}m_{i^{\prime }i},$$

with $N_{i^{\prime }}^{th}$ the number of trees in patch *i′* of area *A_i′_*, calculated using the reference planting density estimated from expert opinion: *μ*^*th*^ = 666 trees.ha^−1^. Thus, the patch of first introduction is selected by choosing a quantile *q_κ_* of the outgoing connectivity calculated beforehand for all patches of the landscape. One can note that the ranking of the patches according to *κ* depends on their respective area and connectivity with neighbouring patches, and that *κ_i′_* reflects the potential of patch *i′* to initiate an epidemic.

### 2.5. Output variable

The age-dependent fruit yield of the hosts in *S* and *E* states in orchard *i* at year *a*, denoted *y_i,a_*, is relative to the maximum yield (i.e. *y_i,a_* is a proportion). The impact of disease spread in the landscape between the beginning of the epidemic (at year *a*_0_) and the end of the simulation (at year *a*_f_) is summarized by the mean equivalent number of fully productive trees per hectare and per year (figure 1):

$$Y=\frac{1}{A_{\mathrm{tot}}.(a_{f}-a_{0}+1)}.\sum_{a=a_{0}}^{a_{f}}\sum_{i}y_{i,a}.(S_{i,a}+E_{i,a}),$$

with *A*_tot_ = 523 ha, the total area covered by the patches.

## 3. Sensitivity analyses

A sensitivity analysis generally consists of four steps: (i) definition of the target parameters and their respective variation ranges and probability distributions; (ii) generation of a numerical experimental design to explore the parameter space; (iii) simulation and (iv) computation of sensitivity indices [5]. These four steps can be complemented with metamodelling, which provides further insights into the relation between the model input and output.

### 3.1. Target parameters and variation ranges

The sensitivity analyses targeted six parameters: the quantile of the outgoing connectivity of the patch of first introduction (*q_κ_*), the probability of introduction (*Φ*), the relative probability of massive introduction (*p*_MI_), the transmission coefficient (*β*), the expected value of the dispersal weighting variable (*W*_exp_) and the expected duration of the latent period (*θ*_exp_).

Only one parameter per epidemiological process was targeted in the sensitivity analyses, in order to easily rank these processes by influence. Moreover, this approach helps target parameters which vary independently from each other. Thus, the beta distribution of the dispersal weighting variable (*W*) was re-parametrized with its expected value, *W*_exp_, and its variance, *W*_var_, in order to vary only *W*_exp_ in the sensitivity analyses (*W*_var_ was kept fixed at its reference value; table 1). The shape parameters of *W* were then calculated by: $s_{1}=(({W_{\exp}}^{2}.(1-W_{\exp}))\text{/}W_{\mathrm{var}})-W_{\exp}$ and $s_{2}=(s_{1}.(1-W_{\exp}))/W_{\exp}$. Similarly, the gamma distribution of the latent period duration was re-parametrized by its expected value, *θ*_exp_, and its variance, *θ*_var_, and only *θ*_exp_ was targeted in the sensitivity analyses. The shape and scale parameters were, respectively, calculated by: $\theta_{1}=(\theta_{\exp}^{2}\text{/}\theta_{\mathrm{var}})$; $\theta_{2}=(\theta_{\mathrm{var}}\text{/}\theta_{\exp})$. Nevertheless, *θ*_var_ was not held constant, because data on incubation periods of many infections show a strong correlation between the median or mean and the standard deviation (electronic supplementary material, Methods S1, table S1 and figure S10). With regard to PPV infection, we obtained a coefficient of 0.35 from the mean and standard deviation estimates of the latent period duration [27]. This is within the range of values for various diseases (electronic supplementary material, table S1) so the variance of the latent period duration was calculated by *θ*_var_ *=* (0.35 *×* *θ*_exp_)^2^.

**Table 1.** Parameters of the epidemiological model: description, reference value and variation ranges of the parameters targeted in each sensitivity analysis.

			broad-range sensitivity analysis		sharka-specific sensitivity analysis	
parameter	description	reference value for sharka	min	max	min	max
*a*_0_	first year of epidemic	1	—	—	—	—
*a_f_*	last year of simulation	35	—	—	—	—
*ψ*	expected orchard duration (years)	15^a^	—	—	—	—
*y*	relative age-dependent yield of hosts in *S* or *E* states	0.00 until 2 years^a^0.50 at 3 years^a^0.65 at 4 years^a^0.85 at 5 years^a^1.00 from 6 to 15 years^a^0.80 from 16 years^a^	—	—	—	—
*q_κ_*	quantile of the outgoing connectivity of the patch of first introduction	0.50	0.00^b^	1.00^b^	0.00^b^	1.00^b^
*ϕ*	probability of introduction at planting	0.007^a^	0.00^b^	1.00^b^	0.0046^a^	0.0107^a^
*ξ*_11_	first shape parameter of the prevalence for small introductions	0.62^a^	—	—	—	—
*ξ*_12_	second shape parameter of the prevalence for small introductions	23.73^a^	—	—	—	—
*ξ*_21_	first shape parameter of the prevalence for massive introductions	1^a^	—	—	—	—
*ξ*_22_	second shape parameter of the prevalence for massive introductions	1^a^	—	—	—	—
*p*_MI_	relative probability of massive introduction	0.00^a^	0.00^b^	1.00^b^	0.00^a^	0.10^a^
*W*_exp_	expected value of the dispersal weighting variable	0.486^c^	0.14^e^	0.86^e^	0.469^c^	0.504^c^
*W*_var_	variance of the dispersal weighting variable	0.0434^c^	—	—	—	—
*β*	transmission coefficient	1.32^d^	0.08^f^	12.8^f^	1.25^d^	1.39^d^
*θ*_exp_	expected duration of the latent period (years)	1.92^d^	0.0027^f,g^	33.00^f^	1.71^d^	2.14^d^
*θ*_var_	variance of the latent period duration (years)	0.44^d^	—	—	—	—

^a^Estimated through expert opinion.

^b^Definition domain.

^c^Estimated in [27] using $h_{j}=(3\text{/}2)\times 1.08^{j-1}$ with *J* = 100.

^d^Estimated in [27].

^e^Definition domain for a unimodal distribution of *W* with *W*_var_ = 0.0434.

^f^See details in electronic supplementary material, Methods S2.

^g^That is, 1 day.

In the broad-range sensitivity analysis, target parameters were varied within their whole definition domain, or, if impossible, were bounded by values below and above which the epidemic process does not fundamentally change any more. Therefore, the variation ranges of *q_κ_*, *Φ* and *p*_MI_ were easily defined by the bounds of their definition domain (i.e. [0; 1]) (table 1). For *W*_exp_ this interval had to be restricted to generate only unimodal beta distributions for *W* (see electronic supplementary material, figure S3C), and thus smooth dispersal kernels (see electronic supplementary material, figure S4). Parameter *θ*_exp_ was bounded by 1 day (i.e. a value close to 0 time step in our model) and 33 years (see electronic supplementary material, Methods S2). At this upper bound, less than 5% (on average) of the hosts infected at planting would become infectious before orchard removal. Finally, bounds for *β* were calculated using a deterministic susceptible-infectious model in a single orchard where only one host was infected at planting (see electronic supplementary material, Methods S2). When *β* was set at its lower bound, only one host was newly infected after 30 years; at its upper bound, 95% of the orchard was infected after 3 years (i.e. when the orchard starts to be productive).

In the sharka-specific sensitivity analysis, the variation ranges of the target parameters were informed by available knowledge on sharka epidemics in French peach orchards (table 1). Previous work enabled the estimation of *β*, *θ*_exp_ and *W*_exp_ [27]; thus, the bounds of these parameters were defined by their 99% credibility intervals. Since the first introduction could occur in any patch of the landscape, *q_κ_* was varied inside its whole definition domain, as in the previous analysis. Bounds of *Φ* and *p*_MI_ were assessed by expert opinion based on the available field data on introductions.

Because *κ* is calculated using *m*, *q_κ_* also depends on *W*_exp_. In order to independently vary *q_κ_* and *W*_exp_, *κ* was calculated using a different connectivity matrix, denoted *m′*, as follows. In the broad-range sensitivity analysis, *m′* was generated using a simple uniform function: $f_{0}(d)=1/(\pi \times 564^{2})$ if d≤564 m; 0 otherwise. Using this function, $\sum_{i\neq i^{\prime }}{m^{\prime }}_{i^{\prime }i}$ describes the proportion of a circular 1 km^2^ zone centred on patch *i′* and occupied by other orchards. In the sharka-specific sensitivity analysis, because *W*_exp_ varied slightly around its reference value, *m′* was generated using the dispersal kernel *f* (see ‘Pathogen dispersal’) with *W*_exp_ = 0.486 (i.e. the reference value). Thanks to these procedures, the six target parameters were sampled independently.

One can note that the variation of some of the target parameters (*p*_MI_, *W*_exp_, *θ*_exp_) led to variation of whole probability distributions (electronic supplementary material, figures S3 and S4) used, in turn, as input in the model.

### 3.2. Calculation of sensitivity indices

Simulations were performed with 15 000 different parameter combinations generated with Sobol's sequences [34,35]. To take stochasticity into account, each combination was replicated 50 times. Let *Y* denote the output variable (i.e. the mean or standard deviation of these replicates), and *X_i_* (*i* = 1, … ,*p*) the input parameters of the model. Sobol's indices are calculated for each *X_i_* as follows:

$${\text{SI}}_{1}^{X_{i}}=\frac{V[E(Y|X_{i})]}{V(Y)}$$

and

$${\text{SI}}_{\mathrm{tot}}^{X_{i}}=\frac{E[V(Y|X_{-i})]}{V(Y)},$$

where *X_-i_* denotes the whole set of parameters except *X_i_*. ${\text{SI}}_{1}^{X_{i}}$ is the first-order index, which measures the main effect of *X_i_* alone; ${\text{SI}}_{\mathrm{tot}}^{X_{i}}$ is the total index, which measures the influence of *X_i_* including all its interactions with other parameters (for details on the computation of Sobol's sensitivity indices, see electronic supplementary material, Methods S3 and [17,18,36]). First-order indices were estimated with Sobol–Saltelli's method [37,38] whereas total indices were estimated with Sobol–Jansen's method [37,39], because these two methods were shown to be good estimators of small first-order indices and (large and small) total indices, respectively. The 95% confidence intervals (CI_95_) of the sensitivity indices were estimated using 10 000 bootstrap replicates [40].

In each sensitivity analysis, key interactions were identified using polynomial regression. A third-degree polynomial including interactions restricted to polynomial terms of degree two was fitted to the means and standard deviations of the model output. Then, the most parsimonious model was obtained using a stepwise selection algorithm based on the Bayesian information criterion (BIC) [41]. Before model fitting, the target parameters were standardized to the mean and standard deviation of their sampling distribution, in order to easily interpret the estimated coefficients as the effect of one standard deviation change in each parameter on the output variable [2]. The first-order sensitivity indices of the metamodel are deduced from Sobol's decomposition (see details in electronic supplementary material, Methods S3):

$${\text{SI}}_{1}^{X_{i}}=\frac{V(b_{0}+b_{i}.Z_{i}+b_{i^{2}}.Z_{i}^{2}+b_{i^{3}}.Z_{i}^{3})}{V(Y)},$$

$${\text{SI}}_{2}^{X_{i}X_{j},\;i\neq j}=\frac{V(b_{i,j}.Z_{i}.Z_{j})}{V(Y)}=\frac{b_{i,j}^{2}}{V(Y)}$$

$$\text{and}\;{\text{SI}}_{3}^{X_{i}X_{j}X_{k},i\neq j\neq k}=\frac{V(b_{i,j,k}.Z_{i}.Z_{j}.Z_{k})}{V(Y)}=\frac{b_{i,j,k}^{2}}{V(Y)},$$

where *Z_i_* is the standardized version of *X_i_*; *b*_0_ denotes the intercept; $b_{i},\;b_{i^{2}}\;\text{and}\;\;b_{i^{3}}$ denote the estimated coefficients of *Z_i_*, $Z_{i}^{2}$, and $Z_{i}^{3}$, respectively; and *b_i,j_* and *b_i,j,k_* denote the estimated coefficients of the products *Z_i_.Z_j_* and *Z_i_.Z_j_.Z_k_*, respectively.

### 3.3. Simulations with fixed parameters

In order to better visualize the impact of the introduction parameters on epidemic spread, 100 simulations were performed for each of the following scenarios:

A. ‘Disease-free’: the pathogen is not introduced in the landscape;
B. ‘Single introduction of one tree’: the pathogen is introduced once at *t* = 1 with only one infected host;
C. ‘Single introduction of several trees’: the pathogen is introduced once at *t* = 1 with possibly several infected hosts;
D. ‘Weakly connected first introduction’: the pathogen is first introduced at *t* = 1 in a weakly connected patch (*q_κ_* = 0.01), and with a risk *Φ* at every subsequent orchard planting;
E. ‘Moderately connected first introduction (reference)’: same as scenario D, but the patch of first introduction is moderately connected (*q_κ_* = 0.50);
F. ‘Highly connected first introduction’: same as scenario D, but the patch of first introduction is highly connected (*q_κ_* = 0.99).

Except the introduction parameters, all the model parameters were fixed at reference values associated with sharka epidemics in French peach orchards, estimated from epidemiological data or expert opinion (table 1). For scenarios C–F, the probability that a tree is infected at introduction is given by *τ*, whose mean is 2.6% (using reference values for *ξ*_11_, *ξ*_12_, *ξ*_21_, *ξ*_22_ and *p*_MI_).

### 3.4. Software

The model was written in R and C languages. Within the R software v. 3.0.3 [42], Sobol's sequences and indices were obtained using the packages *fOptions* (v. 3010.83) and *sensitivity* (v. 1.11), respectively. The stepwise model-selection algorithm used the function *regsubsets* of the package *leaps* (v. 2.9). A simulation takes approximately 4 s with a regular computer (Intel^®^ Core™ i7-4600M). The overall exploration of the model was carried out on a computer cluster, which enabled the parallelization of the 50 stochastic replicates for each sensitivity analysis, each replicate requiring roughly one day to explore the 15 000 parameter combinations.

## 4. Results

### 4.1. Strong influence of latent period duration on the simulation model

**Figure 2.**
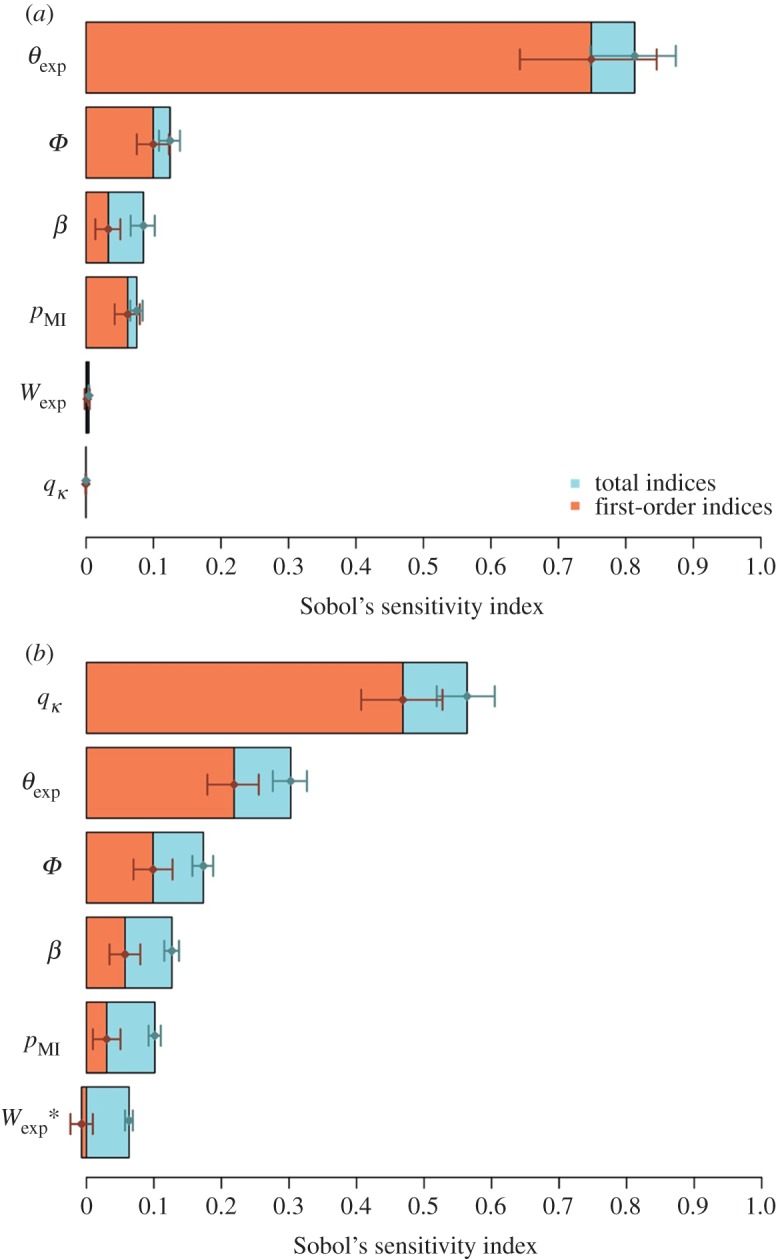
First-order and total Sobol's sensitivity indices of the six target parameters on the mean output (*μ_Y_*, mean number of fully productive trees per hectare and per year) of the stochastic replicates, assessed in the broad-range analysis (*a*, $\Sigma {\mathrm{SI}}_{1}=0.94$), or informed by knowledge acquired on sharka in peach orchards (*b*, $\Sigma {\mathrm{SI}}_{1}=0.87$). *θ*_exp_, expected duration of the latent period; *Φ*, probability of introduction at orchard planting; *β*, transmission coefficient; *p*_MI_, relative probability of massive introduction; *W*_exp_, expected value of the dispersal weighting variable; *q_κ_*, quantile of the outgoing connectivity of the patch of first introduction. Horizontal bars: CI_95_ (assessed using 10 000 bootstrap replicates). * This estimate of the first-order Sobol's index was slightly negative, which may occur when the indices do not significantly differ from 0 [43].

**Figure 3.**
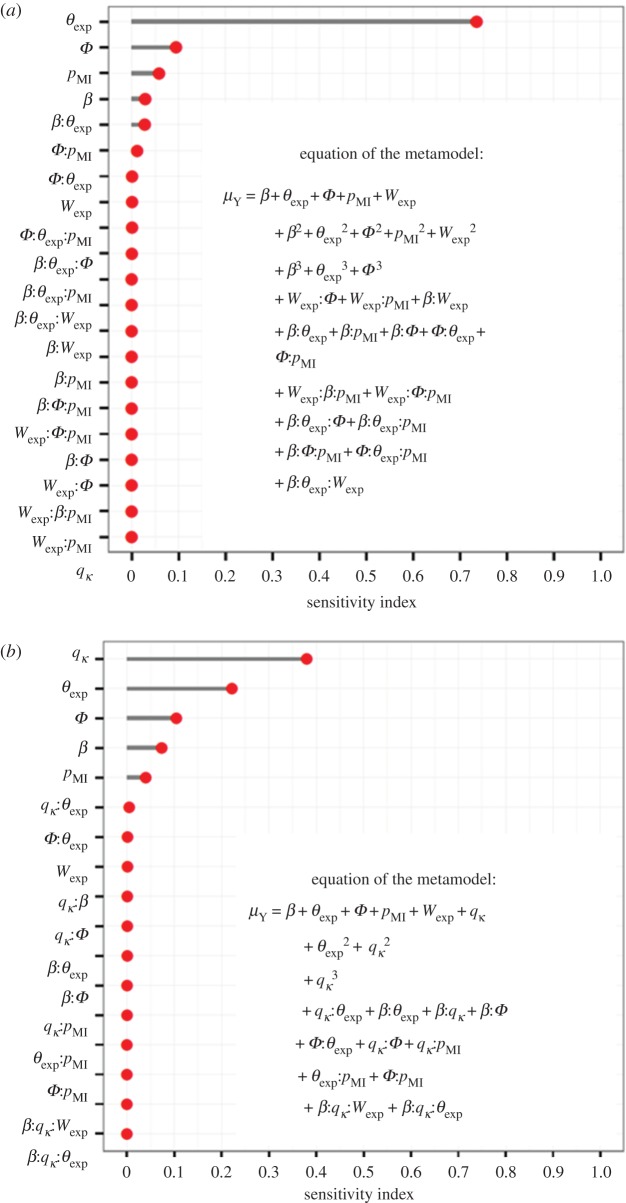
Sensitivity indices of the target parameters and their interactions up to the order 2 on the mean output (*μ_Y_*, mean number of fully productive trees per hectare and per year) of the stochastic replicates, assessed through a degree-3 polynomial optimized using BIC in the broad-range analysis (*a*, *R*^2^ = 0.96), or informed by knowledge acquired on sharka in peach orchards (*b*, *R*^2^ = 0.83). *θ*_exp_, expected duration of the latent period; *Φ*, probability of introduction at orchard planting; *β*, transmission coefficient; *p*_MI_, relative probability of massive introduction; *W*_exp_, expected value of the dispersal weighting variable; *q_κ_*, quantile of the outgoing connectivity of the patch of first introduction.

In the broad-range analysis, the mean output over the stochastic replicates varied from 47 to 567 equivalent fully productive plants per hectare and per year. The sensitivity analysis showed that the expected duration of the latent period (*θ*_exp_) had the strongest impact (figure 2*a*), with a total Sobol's index (SI_tot_) of 0.81 (CI_95_: 0.75–0.87). Next came the probability of introduction (*Φ*), the transmission coefficient (*β*) and the relative probability of massive introduction (*p*_MI_), with SI_tot_ of 0.12 (0.11–0.14), 0.09 (0.07–0.10) and 0.08 (0.07–0.08), respectively. The last two parameters, i.e. the expected value of the dispersal weighting variable (*W*_exp_), and the quantile of the outgoing connectivity of the patch of first introduction (*q_κ_*), had a negligible influence on the output as shown by their very small SI_tot_. The first-order indices (SI_1_) were close to (and mostly not significantly different from) SI_tot_. In addition, the total variability associated with the main effects (assessed by the sum of the SI_1_) explained 94% of the variability in the output. These elements indicate that the simulated process involves few interactions. In order to characterize the relationship between parameters and the mean response, a polynomial metamodel was adjusted to the model output. According to its coefficient estimates, the most important interactions (figure 3*a*) involved firstly *β* and *θ*_exp_ (SI_2_ = 0.03) and, secondly, *Φ* and *p*_MI_ (SI_2_ = 0.01). In addition to the good coefficient of determination (*R*^2^ = 0.96), the goodness-of-fit of this metamodel was confirmed by the estimates of the SI_1_, which did not differ from the indices estimated with Sobol's method by more than 0.01 (figures 2*a* and 3*a*).

**Figure 4.**
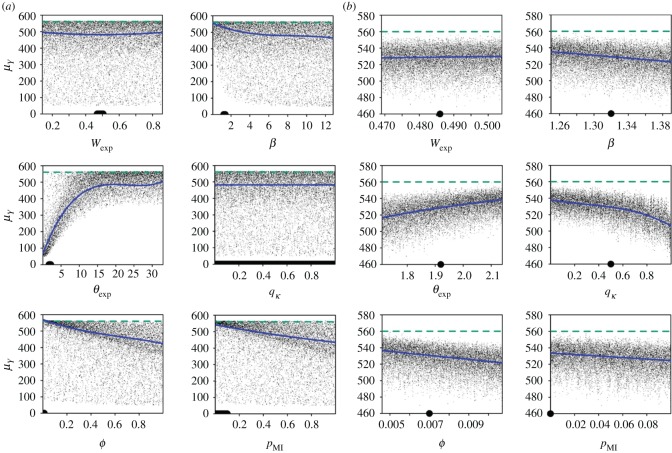
Effect of each target parameter on the mean output (*μ_Y_*, mean number of fully productive trees per hectare and per year) in the broad-range analysis (*a*), or informed by knowledge acquired on sharka in peach orchards (*b*). Each point represents the mean of 50 stochastic replicates of model simulations performed with the same combination of parameters. Blue line, prediction using the degree-3 polynomial optimized using BIC (all other parameters fixed at their mean value); this line is extremely close to the moving average of the number of productive trees, which has not been represented for legibility. Green dashed line: number of productive trees in the disease-free scenario (i.e. 565). In (*a*) the thick line on the *x*-axis corresponds to the variation range used in the sharka-specific analysis; in (*b*) the full circle on the *x*-axis indicates the reference value of each parameter. *W*_exp_, expected value of the dispersal weighting variable (higher values correspond to longer dispersal distances); *β*, transmission coefficient; *θ*_exp_, expected duration of the latent period; *q_κ_*, quantile of the outgoing connectivity of the patch of first introduction; *Φ*, probability of introduction at orchard planting; *p*_MI_, relative probability of massive introduction.

The estimated metamodel was useful to predict the mean number of productive plants for each value of the target parameters (all other parameters being fixed at their mean value, figure 4*a*). The number of productive plants greatly increased with the duration of the latent period, until approaching the maximum (i.e. 565 fully productive plants per hectare and per year, in the absence of disease) for expected durations of the latent period above 15 years. Because this saturation phenomenon occurred for half of the simulations, graphics associated with the other parameters showed a lot of noise. Despite this noise, it is possible to see that increasing values of *β*, *Φ* and *p*_MI_ had a negative impact on the output, whereas *W*_exp_ and *q_κ_* had a negligible effect, as already indicated by the sensitivity indices.

The analysis performed on the standard deviation of the output revealed that the model stochasticity was mainly due to many interactions of low influence (except the one between *θ*_exp_ and *Φ*), as shown by the low values of SI_1_ and of the interaction indices calculated through the metamodel (see electronic supplementary material, figures S5*a* and S6*a*). The model output was more variable when the latent period duration and the probability of introduction were low (see electronic supplementary material, figure S7a).

### 4.2. Importance of the patch of first introduction for sharka epidemics

In the analysis specific to sharka epidemics in peach orchards, the number of productive trees varied from 469 to 559 per hectare and per year. Parameter *q_κ_* was the most influential, with an SI_tot_ of 0.56 (0.52–0.60). Parameters *θ*_exp_, *Φ*, *β, p*_MI_ and *W*_exp_ followed with SI_tot_ of 0.30 (0.28–0.33), 0.17 (0.16–0.19), 0.13 (0.12–0.14), 0.10 (0.09–0.11) and 0.06 (0.06–0.07), respectively. Interactions had a small effect, the main effects of the target parameters explaining 87% of the variability in the mean number of productive trees (figure 2*b*). These results were similar to the indices calculated through the metamodel (SI_1_ did not differ from Sobol's indices by more than 0.09). The main (although weak) interaction was between *θ*_exp_ and *q_κ_*, with an SI_2_ of 0.005 (figure 3*b*, *R*^2^ = 0.83). Similarly to the broad-range analysis, the graphical representation of the influence of each target parameter on the output showed that the epidemic impact increased when *q_κ_*, *Φ*, *p*_MI_ and *β* increased, and when *θ*_exp_ decreased (figure 4*b*).

Stochasticity in the sharka-specific analysis was due to the main effects of *θ*_exp_, *q_κ_*, *p*_MI_ and *β*, and to many interactions, as revealed by the large differences between SI_tot_ and the SI_1_ for the six target parameters. Although these interactions were numerous, the metamodel showed that each one had a small sensitivity index (see electronic supplementary material, figure S5*b* and S6*b*). The number of productive trees was more variable when *q_κ_*, *p*_MI_ and *β* increased, and when *θ*_exp_ decreased, i.e. when the epidemic was stronger (see electronic supplementary material, figure S7*b*).

### 4.3. Simulation of different epidemic scenarios

**Figure 5.**
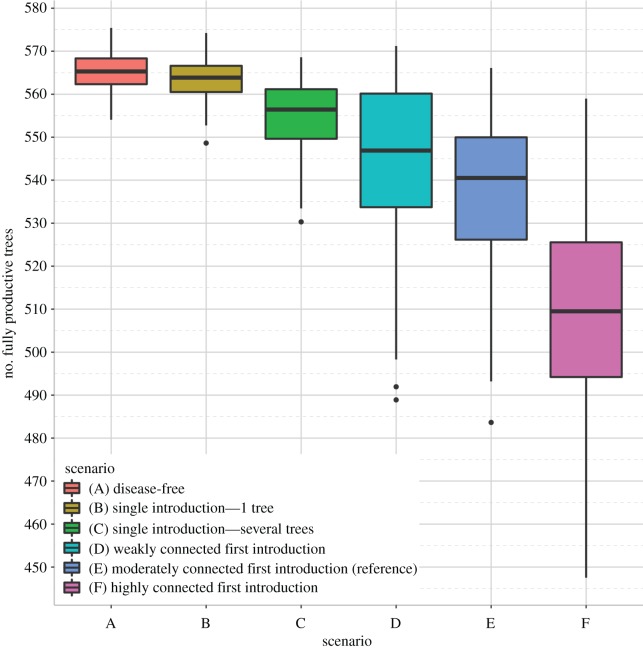
Distribution of the number of fully productive trees per hectare and per year after 35 years of simulation (*Y*) under six scenarios. (A) The pathogen is not introduced in the landscape. (B) The pathogen is introduced once at *t* = 1 in a moderately connected patch, with only one infected host. (C) The pathogen is introduced once at *t* = 1 in a moderately connected patch, with possibly several infectious trees (mean prevalence of 2.55%). (D) The pathogen is first introduced at *t* = 1 in a weakly connected patch, and at every subsequent orchard planting in the landscape with a risk of 0.7%, and with possibly several infectious trees (mean prevalence of 2.55%) for each introduction. (E) Same as (D) but the patch of first introduction is moderately connected. (F) Same as (D) but the patch of first introduction is highly connected. Except the introduction parameters, all the model parameters were fixed at their reference values (table 1).

Because the sharka-specific sensitivity analysis highlighted the role of introductions in epidemic spread, several epidemic scenarios were simulated to illustrate the impact of introduction parameters. In the absence of disease, a median number of 565 (558–572) fully productive trees were grown per hectare and per year (figure 5, scenario A). The number of productive trees was almost the same, 564 (554–572), when PPV was introduced only once through the planting of one infectious tree (scenario B). Epidemics slightly impacted the number of productive trees (556; CI_95_ = 538–568) when several infected trees were allowed to be planted (scenario C), showing the importance of prevalence at introduction. In more realistic scenarios, corresponding to repeated introductions of PPV [27], the mean number of fully productive trees dropped to 547 (499–568), 541 (498–564) and 509 (462–551) when the first introduction occurred in a weakly (scenario D), moderately (scenario E) or highly (scenario F) connected patch, respectively. These results illustrate not only the impact of multiple introductions on sharka epidemic spread, but also and most importantly of the location of the first introduction. Videos of epidemic spread in the landscape in scenarios B to F are available as electronic supplementary material, Videos S1–S5.

## 5. Discussion

### 5.1. Sobol's method and metamodelling complement each other

The first aim of this study was to investigate the combined use of Sobol's method [44,45] and polynomial metamodelling to identify key factors and interactions in a stochastic spatiotemporal model simulating epidemics among host plants. The parameter space was explored through a quasi Monte Carlo sampling, generated using Sobol's sequences because of their good space-filling and fast convergence properties. Sobol's method to calculate sensitivity indices benefited from numerous improvements of its precision and computational cost [37–39,44,46,47]. In our work, Sobol's method proved to be an efficient tool to rank parameters by influence on the output variable, and thus to identify potential targets for further research or disease management. Additionally, in the case of large differences between first-order and total sensitivity indices, this method reveals the importance of interactions, which must be further investigated for a careful management. In our approach, Sobol's method and polynomial metamodelling complemented each other. Both methods gave extremely close estimates of the first-order sensitivity indices. Sobol's method is more straightforward in the estimation of the total indices, as well as the CIs (figure 2). Polynomial metamodelling helped identify specifically which interactions are involved in the epidemic process, and visualize model behaviour in response to the variation of input parameters (figure 4).

The approach can be applied to many simulation models, and is especially useful for stochastic models. To account for stochasticity, some authors replicated simulations for a few selected parameter combinations to characterize the variance or the distribution of the output variable [48,49]. In another approach, every parameter combination of the simulation design was replicated in such way that an extra sensitivity index could be calculated by ANOVA to describe the proportion of the total variability attributable to the variability among parameter combinations [8,15]. Here, we adopted an approach developed originally with non-spatial epidemic models [7,29]. Stochasticity was accounted for by replicating simulations for each parameter combination and by performing sensitivity analyses on the mean and standard deviation of the replicates. However, the total number of simulations (including the different parameter combinations and the stochastic replicates per combination) grows as a power of the number of parameters targeted in the sensitivity analysis. This curse of dimensionality necessitates a compromise between the resolution of parameter space exploration, the precision of the model output and the total computational cost [37,50]. In this study, the parameter space was thoroughly explored using 15 000 different parameter combinations, and each combination was replicated 50 times because this number was largely sufficient to get robust estimates of the mean and standard deviation associated with each combination (not shown).

### 5.2. Impact of the variation range of the target parameters

Our second aim was to uncover the key drivers of plant epidemics, using sensitivity analysis. The choice of variation ranges for the target parameters has a great impact on the results of such analysis. In the first sensitivity analysis, we used the broadest possible ranges of values for the target parameters, ignoring any prior information. This analysis is thus indicative of the relative intrinsic influence of the epidemiological processes on model behaviour in the absence of knowledge on parameter values. Our results showed that the latent period duration is the most influential parameter of the model. Depending on the latent period duration, the impact of the simulated epidemic may be completely different, from insignificant and stable (when the latent period lasts more than 15 years) to important and highly variable (if it lasts less than 5 years). It is very likely that the threshold distinguishing insignificant epidemics from others (i.e. approximately 15 years) is linked to the mean orchard duration. In order to test whether the high influence of the latent period in the broad-range sensitivity analysis was just due to its very wide variation range, we replicated this analysis with smaller upper bounds for the mean duration of the latent period (from 5 to 33 years, see electronic supplementary material, figure S8). This additional information demonstrates that the results are qualitatively robust to changes in this upper bound. One may also note that the most significant interaction involves the mean latent period and the transmission coefficient (figure 3*a*), which may explain why the latter becomes the most influential parameter for epidemics whose latent periods are below 7 years (see electronic supplementary material, figure S8).

In the second sensitivity analysis, the variation range of all target parameters focused on the best available values regarding sharka epidemiology in peach orchards. These variation ranges defined a parameter space which had been poorly explored in the previous sensitivity analysis (figure 4*a*, thick line on the *x*-axis), in spite of the large number of tested parameter combinations. Within this sharka-specific analysis, whatever the parameter combination the mean equivalent number of productive trees was significantly lower than what could be obtained in the absence of disease. This encourages the application of control measures. Here, in contrast with the broad-range analysis, we show that the connectivity of the patch of introduction is the most influential parameter on sharka epidemics. Consequently, the outgoing connectivity of a given patch of the landscape can be considered as an index of the risk to initiate an epidemic, and sharka control may include for example preventive and regular surveys of these patches.

Because this index of epidemic risk depends on patch connectivity, it was surprising to observe a low influence of the dispersal kernel (represented by the parameter *W*_exp_) in both the broad-range and sharka-specific analyses. On the contrary, mean dispersal distance was shown to be a key driver of spread of fungal pathogens [14] and invasive plants [10]. In our broad-range analysis, one may think that the low impact of the dispersal kernel might be due to the fact that the variance of the weighting variable (*W*_var_) was held constant, which may result in dispersal kernels with similar shapes. Nevertheless, variation of the dispersal kernel covered a wide range of distances (see electronic supplementary material, figure S4). Thus, another explanation may be the patchiness of the real agricultural landscape that we used for the simulations (see electronic supplementary material, figure S1). Indeed, short-distance dispersal resulted in very slow disease spread, and long-distance dispersal resulted in a higher proportion of dispersal events ending outside the orchards (where hosts are absent). This is an interesting property of fragmented agricultural landscapes. In the sharka-specific analysis, the low influence of the dispersal kernel can be explained in part by the narrow range of variation in *W*_exp_. To investigate the impact of the parameter variation ranges, we performed a sensitivity analysis similar to the sharka-specific analysis except that *W*_exp_ varied as in the broad-range analysis. This resulted in a very high sensitivity index for *W*_exp_ (see electronic supplementary material, figure S9). This exemplifies how the sensitivity indices depend on the extent of the variation ranges of the target parameters. As another illustration of the influence that the variation range of a parameter can have, a model simulating *tomato yellow leaf curl virus* (TYLCV) epidemics was almost insensitive to the latent period duration [11], whereas it was the first and the second most influential parameter in our broad-range and sharka-specific analyses, respectively. This difference can be explained by the fact that our output variable (the number of productive hosts) included latently infected hosts whereas the output in [11] did not, but also by the fact that the variation range of the latent period duration was narrower in the TYLCV study. These elements, and the strong differences between the two analyses performed in the present study, highlight the importance of identifying appropriate ranges of variation for the target parameters in a sensitivity analysis. In particular, our results show that the parameters with the greatest intrinsic influence on overall behaviour of the model are not necessarily the key drivers of epidemic spread in a specific situation. Therefore, in different pathosystems and epidemiological contexts, the variation ranges, and especially their width, must be re-examined in order to identify relevant targets for research efforts and management strategies.

### 5.3. Genericity of the model

In this study, an existing spatiotemporal stochastic model of sharka spread, based on an SEIR architecture, was modified in order to perform sensitivity analyses. This model corresponds to vector-borne diseases of perennial plants (small turnover of crops, long latent period, pathogen introductions at crop planting), but its parametrization and flexibility enable the study of various horizontally transmitted plant infections, including wind-dispersed epidemics of annual crops. Nevertheless, some assumptions of the model are specific to sharka and should be re-examined for other diseases. In particular, for vector-borne diseases transmitted in a persistent manner, infectious vectors are able to transmit the pathogen to several healthy hosts. Thus, modelling such pathosystem needs to account for vector movement throughout their infectious period, which generally does not start immediately after acquisition. The resulting dispersal function may thus have a completely different shape. We also considered that latently infected hosts were still fully productive, whereas infectious individuals had a null productivity (no yield, or sales ban), and that the disease did not trigger host death (which influences the duration of the infectious period); such assumptions do not apply to all diseases.

Our study uncovered the key role played by highly connected introduction patches on sharka spread. This result highlights that paying close attention to these patches might prevent serious epidemics. Our next aims are to enrich this model with various control strategies and simulate various realistic landscapes in order to more directly help improve disease management.

## Supplementary Material

Supporting Information

## Data accessibility

Data and code are available from the Dryad Digital Repository: http://dx.doi.org/10.5061/dryad.b8kb0 [51].

## References

[1] Gilligan CA 2008 Sustainable agriculture and plant diseases: an epidemiological perspective. Phil. Trans. R. Soc. B 363, 741–759. (doi:10.1098/rstb.2007.2181) 17827101 10.1098/rstb.2007.2181 PMC2610107

[2] Coutts SR, Yokomizo H 2014 Meta-models as a straightforward approach to the sensitivity analysis of complex models. Popul. Ecol. 56, 7–19. (doi:10.1007/s10144-013-0422-1)

[3] Fraser C, Riley S, Anderson RM, Ferguson NM 2004 Factors that make an infectious disease outbreak controllable. Proc. Natl Acad. Sci. USA 101, 6146–6151. (doi:10.1073/pnas.0307506101) 15071187 10.1073/pnas.0307506101 PMC395937

[4] Bassanezi RB, Montesino LH, Gimenes-Fernandes N, Yamamoto PT, Gottwald TR, Amorim L, Filho AB 2012 Efficacy of area-wide inoculum reduction and vector control on temporal progress of huanglongbing in young sweet orange plantings. Plant Dis. 97, 789–796. (doi:10.1094/PDIS-03-12-0314-RE) 10.1094/PDIS-03-12-0314-RE 30722592

[5] Faivre R, Ioos B, Mahévas S, Makowski D, Monod H 2013 Analyse de Sensibilité et Exploration de Modèles, 324p Versailles, France: Editions Quae.

[6] Saltelli A, Tarantola S, Campolongo F 2000 Sensitivity analysis as an ingredient of modeling. Stat. Sci. 15, 377–395. (doi:10.1214/ss/1009213004)

[7] Courcoul A, Monod H, Nielen M, Klinkenberg D, Hogerwerf L, Beaudeau F, Vergu E 2011 Modelling the effect of heterogeneity of shedding on the within herd *Coxiella burnetii* spread and identification of key parameters by sensitivity analysis. J. Theor. Biol. 284, 130–141. (doi:10.1016/j.jtbi.2011.06.017) 21723294 10.1016/j.jtbi.2011.06.017

[8] Lurette A, Touzeau S, Lamboni M, Monod H 2009 Sensitivity analysis to identify key parameters influencing *Salmonella* infection dynamics in a pig batch. J. Theor. Biol. 258, 43–52. (doi:10.1016/j.jtbi.2009.01.026) 19490864 10.1016/j.jtbi.2009.01.026

[9] Barclay HJ, Vreysen MJB 2011 A dynamic population model for tsetse (Diptera: Glossinidae) area-wide integrated pest management. Popul. Ecol. 53, 89–110. (doi:10.1007/s10144-010-0224-7)

[10] Coutts SR, van Klinken RD, Yokomizo H, Buckley YM 2011 What are the key drivers of spread in invasive plants: dispersal, demography or landscape: and how can we use this knowledge to aid management? Biol. Invasions 13, 1649–1661. (doi:10.1007/s10530-010-9922-5)

[11] Holt J, Colvin J, Muniyappa V 1999 Identifying control strategies for tomato leaf curl virus disease using an epidemiological model. J. Appl. Ecol. 36, 625–633. (doi:10.1046/j.1365-2664.1999.00432.x)

[12] Jeger MJ, Chan MS 1995 Theoretical aspects of epidemics: uses of analytical models to make strategic management decisions. Can. J. Plant. Pathol. 17, 109–114. (doi:10.1080/07060669509500701)

[13] Chan MS, Jeger MJ 1994 An analytical model of plant virus disease dynamics with roguing and replanting. J. Appl. Ecol. 31, 413–427. (doi:10.2307/2404439)

[14] Xu XM, Ridout MS 1998 Effects of initial epidemic conditions, sporulation rate, and spore dispersal gradient on the spatio-temporal dynamics of plant disease epidemics. Phytopathology 88, 1000–1012. (doi:10.1094/PHYTO.1998.88.10.1000) 18944811 10.1094/PHYTO.1998.88.10.1000

[15] Papaïx J, Adamczyk-Chauvat K, Bouvier A, Kiêu K, Touzeau S, Lannou C, Monod H 2014 Pathogen population dynamics in agricultural landscapes: the *Ddal* modelling framework. Infect. Genet. Evol. 27, 509–520. (doi:10.1016/j.meegid.2014.01.022) 24480053 10.1016/j.meegid.2014.01.022

[16] Morris MD 1991 Factorial sampling plans for preliminary computational experiments. Technometrics 33, 161–174. (doi:10.2307/1269043)

[17] Saltelli A, Chan K, Scott EM 2000 Sensitivity analysis, p. 475 London, UK: Wiley.

[18] Saltelli A, Ratto M, Andres T, Campolongo F, Cariboni J, Gatelli D, Saisana M, Tarantola S 2008 Global sensitivity analysis: the primer, 304 p New York, NY: Wiley.

[19] Cambra M, Capote N, Myrta A, Llácer G 2006 *Plum pox virus* and the estimated costs associated with sharka disease. EPPO Bull. 36, 202–204. (doi:10.1111/j.1365-2338.2006.01027.x)

[20] Rimbaud L, Dallot S, Gottwald T, Decroocq V, Jacquot E, Soubeyrand S, Thébaud G 2015 Sharka epidemiology and worldwide management strategies: learning lessons to optimize disease control in perennial plants. Annu. Rev. Phytopathol. 53, 357–378. (doi:10.1146/annurev-phyto-080614-120140) 26047559 10.1146/annurev-phyto-080614-120140

[21] Németh MV 1986 Plum pox (Sharka). In Virus, mycoplasma and rickettsia diseases of fruit trees (ed. MV Németh), pp. 463–479. Budapest, Hungary: Akademiai Kiaido.

[22] Gildow F, Damsteegt V, Stone A, Schneider W, Luster D, Levy L 2004 Plum pox in North America: identification of aphid vectors and a potential role for fruit in virus spread. Phytopathology 94, 868–874. (doi:10.1094/Phyto.2004.94.8.868) 18943108 10.1094/PHYTO.2004.94.8.868

[23] Labonne G, Yvon M, Quiot J-B, Avinent L, Llacer G 1995 Aphids as potential vectors of plum pox virus: comparison of methods of testing and epidemiological consequences. Acta Hortic. 386, 207–218. (doi:10.17660/ActaHortic.1995.386.27)

[24] Harris KF 1977 An ingestion-egestion hypothesis of noncirculative virus transmission. In Aphids as virus vectors (eds Harris KF, Maramorosch K), pp. 165–220. London, CA: Academic Press.

[25] Rimbaud L, Dallot S, Delaunay A, Borron S, Soubeyrand S, Thébaud G, Jacquot E 2015 Assessing the mismatch between incubation and latent periods for vector-borne diseases: the case of sharka. Phytopathology 105, 1408–1416. (doi:10.1094/PHYTO-01-15-0014-R) 26512749 10.1094/PHYTO-01-15-0014-R

[26] Rimbaud L, Delaunay A, Soubeyrand S, Jacquot E, Thébaud G 2015 Model-based optimization of an experimental protocol to assess the mismatch between incubation and latency periods for *Plum pox virus*. Acta Hortic. 1063, 159–166. (doi:10.17660/ActaHortic.2015.1063.22)

[27] Pleydell DRJ, Soubeyrand S, Dallot S, Labonne G, Chadœuf J, Jacquot E, Thébaud G 2017 Estimation of the dispersal distances of an aphid-borne virus in a patchy landscape. bioRxiv 109561 (doi:10.1101/109561)

[28] Klein EK, Lavigne C, Picault H, Renard M, Gouyon P-H 2006 Pollen dispersal of oilseed rape: estimation of the dispersal function and effects of field dimension. J. Appl. Ecol. 43, 141–151. (doi:10.1111/j.1365-2664.2005.01108.x)

[29] Fabre F, Bruchou C, Palloix A, Moury B 2009 Key determinants of resistance durability to plant viruses: insights from a model linking within- and between-host dynamics. Virus Res. 141, 140–149. (doi:10.1016/j.virusres.2008.11.021) 19159653 10.1016/j.virusres.2008.11.021

[30] Fabre F, Rousseau E, Mailleret L, Moury B 2012 Durable strategies to deploy plant resistance in agricultural landscapes. New Phytol. 193, 1064–1075. (doi:10.1111/j.1469-8137.2011.04019.x) 22260272 10.1111/j.1469-8137.2011.04019.x

[31] Quiot J-B, Labonne G, Boeglin M, Adamolle C, Renaud LY, Candresse T 1995 Behaviour of two isolates of plum pox virus inoculated on peach and apricot trees: first results. Acta Hortic. 386, 290–298. (doi:10.17660/ActaHortic.1995.386.39)

[32] Sutic D 1971 Etat des recherches sur le virus de la sharka. Ann. Phytopathol. (special issue), 161–170.

[33] Bouvier A, Kiêu K, Adamczyk K, Monod H 2009 Computation of the integrated flow of particles between polygons. Environ. Model. Softw. 24, 843–849. (doi:10.1016/j.envsoft.2008.11.006)

[34] Sobol’ IM 1967 On the distribution of points in a cube and the approximate evaluation of integrals. USSR Comput. Math. Math. Phys. 7, 86–112. (doi:10.1016/0041-5553(67)90144-9)

[35] Sobol IM 1976 Uniformly distributed sequences with an additional uniform property. USSR Comput. Math. Math. Phys. 16, 236–242. (doi:10.1016/0041-5553(76)90154-3)

[36] Sobol IM 1993 Sensitivity analysis for non-linear mathematical models. Math. Modell. Comput. Exp. 1, 407–414.

[37] Saltelli A, Annoni P, Azzini I, Campolongo F, Ratto M, Tarantola S 2010 Variance based sensitivity analysis of model output: design and estimator for the total sensitivity index. Comput. Phys. Commun. 181, 259–270. (doi:10.1016/j.cpc.2009.09.018)

[38] Sobol’ IM, Tarantola S, Gatelli D, Kucherenko SS, Mauntz W 2007 Estimating the approximation error when fixing unessential factors in global sensitivity analysis. Reliab. Eng. Syst. Safe 92, 957–960. (doi:10.1016/j.ress.2006.07.001)

[39] Jansen MJW 1999 Analysis of variance designs for model output. Comput. Phys. Commun. 117, 35–43. (doi:10.1016/S0010-4655(98)00154-4)

[40] Archer GEB, Saltelli A, Sobol IM 1997 Sensitivity measures, ANOVA-like techniques and the use of bootstrap. J. Stat. Comput. Simul. 58, 99–120. (doi:10.1080/00949659708811825)

[41] Schwarz G 1978 Estimating the dimension of a model. Ann. Stat. 6, 461–464. (doi:10.1214/aos/1176344136)

[42] R Core Team. 2012 R: A language and environment for statistical computing, p. 3551 Vienna, Austria: R Foundation for Statistical Computing.

[43] Glen G, Isaacs K 2012 Estimating Sobol sensitivity indices using correlations. Environ. Model. Softw. 37, 157–166. (doi:10.1016/j.envsoft.2012.03.014)

[44] Saltelli A 2002 Making best use of model evaluations to compute sensitivity indices. Comput. Phys. Commun. 145, 280–297. (doi:10.1016/S0010-4655(02)00280-1)

[45] Sobol’ IM 2001 Global sensitivity indices for nonlinear mathematical models and their Monte Carlo estimates. Math. Comput. Simul. 55, 271–280. (doi:10.1016/S0378-4754(00)00270-6)

[46] Janon A, Klein T, Lagnoux A, Nodet M, Prieur C 2014 Asymptotic normality and efficiency of two Sobol index estimators. ESAIM Probab. Stat. 18, 342–364. (doi:10.1051/ps/2013040)

[47] Monod H, Naud C, Makowski D 2006 Uncertainty and sensitivity analysis for crop models. In Working with dynamic crop models: evaluation, analysis parameterization, and applications (eds Wallach D, Makowski D, Jones JW), pp. 55–100. Amsterdam, Netherlands: Elsevier Science Biomedical Division.

[48] Damiani C, Filisetti A, Graudenzi A, Lecca P 2013 Parameter sensitivity analysis of stochastic models: application to catalytic reaction networks. Comput. Biol. Chem. 42, 5–17. (doi:10.1016/j.compbiolchem.2012.10.007) 23246776 10.1016/j.compbiolchem.2012.10.007

[49] Ginot V, Gaba S, Beaudouin R, Aries F, Monod H 2006 Combined use of local and ANOVA-based global sensitivity analyses for the investigation of a stochastic dynamic model: application to the case study of an individual-based model of a fish population. Ecol. Model. 193, 479–491. (doi:10.1016/j.ecolmodel.2005.08.025)

[50] Marrel A, Iooss B, Da Veiga S, Ribatet M 2012 Global sensitivity analysis of stochastic computer models with joint metamodels. Stat. Comput. 22, 833–847. (doi:10.1007/s11222-011-9274-8)

[51] Rimbaud L, Bruchou C, Dallot S, Pleydell DRJ, Jacquot E, Soubeyrand S, Thébaud G 2018 Data from: Using sensitivity analysis to identify key factors for the propagation of a plant epidemic Dryad Digital Repository (http://dx.doi.org/10.5061/dryad.b8kb0) 10.1098/rsos.171435 PMC5792923 29410846

