# Supplementary material for: Using sensitivity analysis to identify key factors for the propagation of a plant epidemic
Source: R Soc Open Sci. 2018 Jan 17;5(1):171435. doi: 10.1098/rsos.171435 (PMC5792923; doi:10.1098/rsos.171435)
Supplement: Supporting Information [file rsos171435supp1.pdf]

Supporting Information

Article title: Using sensitivity analysis to identify key factors for the propagation of a plant epidemic

Authors: Loup Rimbaud, Claude Bruchou, Sylvie Dallot, David R. J. Pleydell, Emmanuel Jacquot, Samuel Soubeyrand and Gaël Thébaud

The following Supporting Information is available for this article:

Figure S1. Landscape used in this study

Figure S2. Computation of the weights of the exponential dispersal functions

Figure S3. Variation of probability distributions used as input in the model

Figure S4. Variation of the dispersal kernel and of the mean dispersal distance

Figure S5. Sobol's sensitivity indices for the standard deviation of the output

Figure S6. Sensitivity indices of the target parameters and their interactions for the standard deviation of the output

Figure S7. Effect of each target parameter on the standard deviation of the output

Figure S8. Robustness of the broad-range sensitivity analysis to changes in the upper bound of the expected duration of the latent period

Figure S9. Sharka-specific sensitivity analysis performed with a wide variation range for the dispersal kernel

Methods S1. Mean and standard deviation of the latent period duration

Methods S2. Parameter bounds in the broad-range sensitivity analysis

Methods S3. Sobol's sensitivity indices

Video S1. scenario B) Single PPV introduction through 1 infectious tree in a moderately connected patch

Video S2. scenario C) Single PPV introduction through several infectious trees in a moderately connected patch.

Video S3. scenario D) Multiple PPV introductions through several infectious trees, with 1st introduction in a weakly connected patch

Video S4. scenario E) Multiple PPV introductions through several infectious trees, with 1st introduction in a moderately connected patch (reference scenario)

Video S5. scenario F) Multiple PPV introductions through several infectious trees, with 1st introduction in a highly connected patch

Figure S1. Landscape used in this study, composed of 553 patches in a peach-growing area in south-eastern France. It roughly consists in a 4.5×4.5 km square, with a total area of 524 ha (average patch area of 0.95 ha).

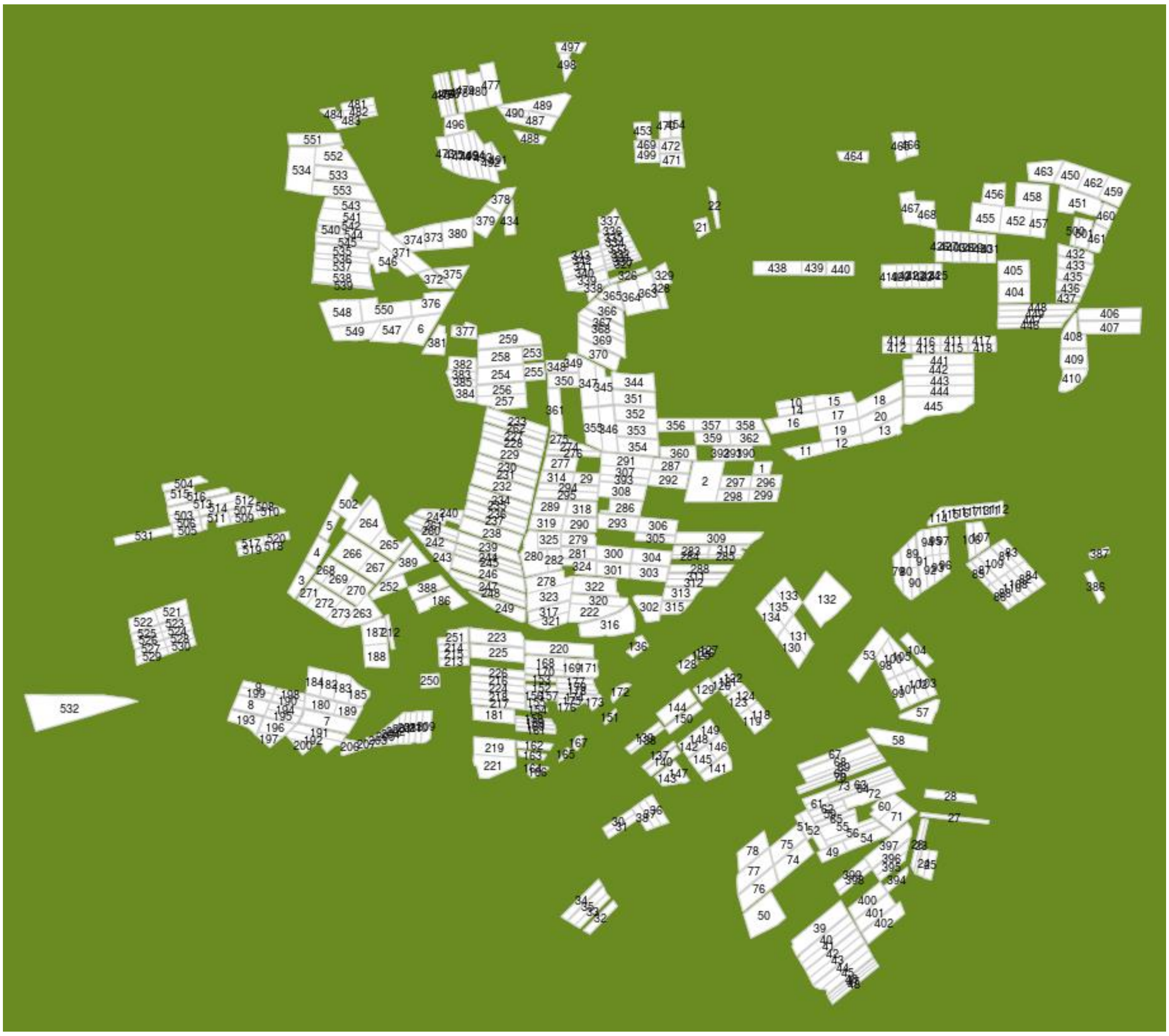

Figure S2. Computation of the weights of the exponential dispersal functions. The weights are computed from the integration of the probability density function of the dispersal weighting variable (W) on J=20 sub-intervals of [0; 1]. In this example, W_{exp}=0.486 and W_{var}=0.0434.

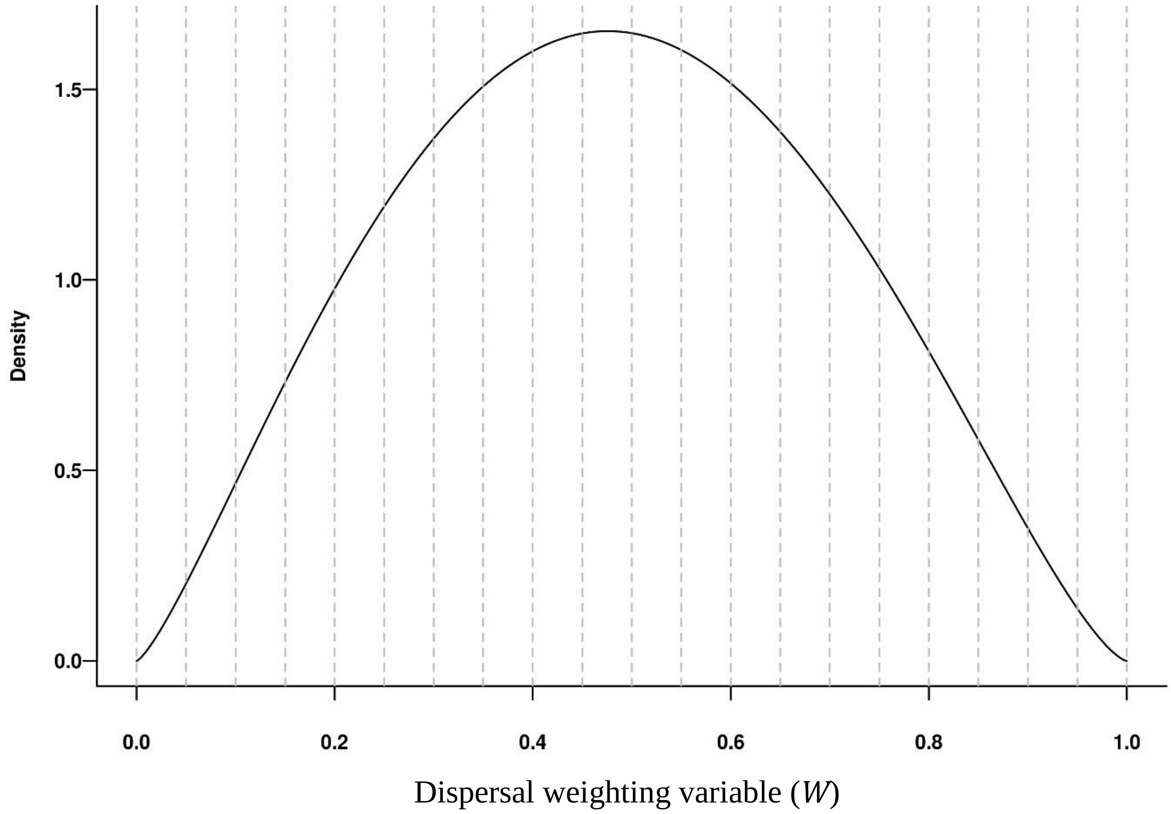

Figure S3. Variation of probability distributions used as input of the model in the broad-range (a-c-e) and the sharka-specific (b-d-f) sensitivity analyses. a-b: Distribution of the probability for a tree to be infected in an orchard contaminated at planting (τ, mixture of beta distributions) for different values of the relative probability of massive introduction (p_{MI}). c-d: Probability distribution of the dispersal weighting variable (W, beta distribution) for different expected values of the dispersal weighting variable (W_{exp}). e-f: Probability distribution of the latent period duration (gamma distributions) for different expected durations of the latent period (θ_{exp}).

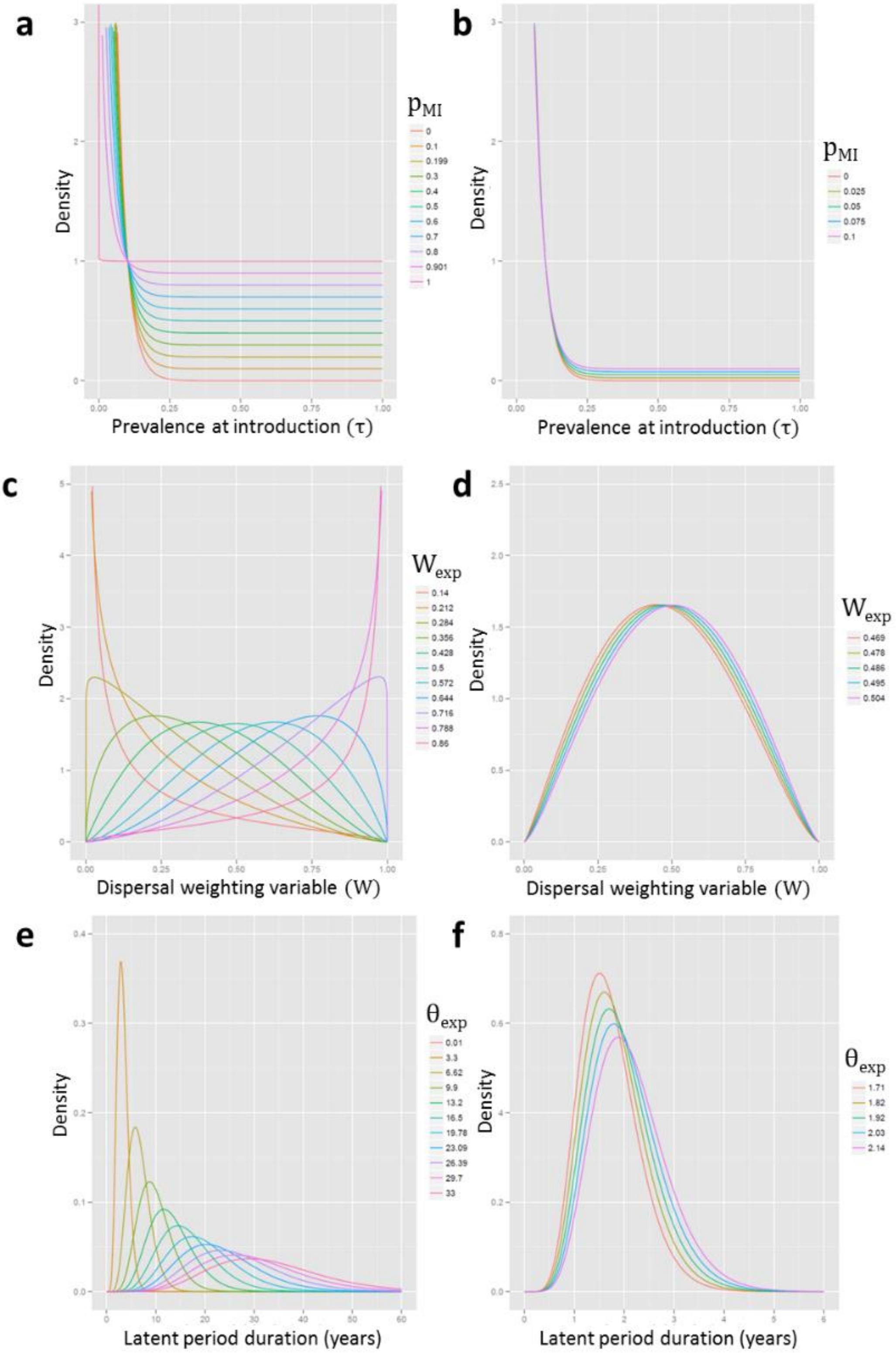

Figure S4. Variation of the mean dispersal distance in the broad-range sensitivity analysis**,** for different expected values of the dispersal weighting variable (W_{exp}). Inset: the corresponding dispersal kernels (mixture of exponential). The colour code matches that of Figure S1c.

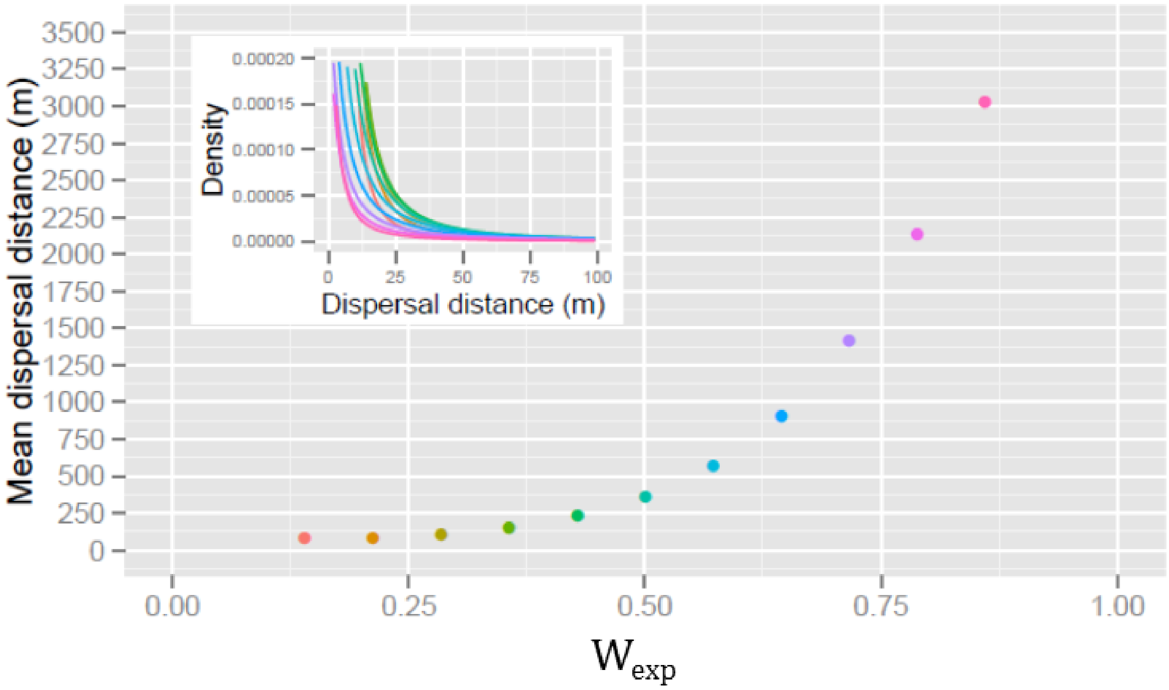

Figure S5. First-order and total Sobol's sensitivity indices of the 6 target parameters on the standard deviation of the output (σ_Y, standard deviation of the equivalent number of fully productive trees per hectare and per year) of the stochastic replicates, assessed in the broad-range analysis (a, $\sum SI_1 = 0.32$), or informed by knowledge acquired on sharka in peach orchards (b, $\sum SI_1 = 0.49$). θ_{exp}: expected duration of the latent period; Φ: probability of introduction at orchard planting; β: transmission coefficient; p_{MI}: relative probability of massive introduction; W_{exp}: expected value of the dispersal weighting variable; q_K: quantile of the outgoing connectivity of the patch of first introduction. Horizontal bars: CI_{95} (assessed using 10,000 bootstrap replicates). *: the estimates of the 1st-order Sobol indices were slightly negative, which may occur when the indices do not significantly differ from 0 [49].

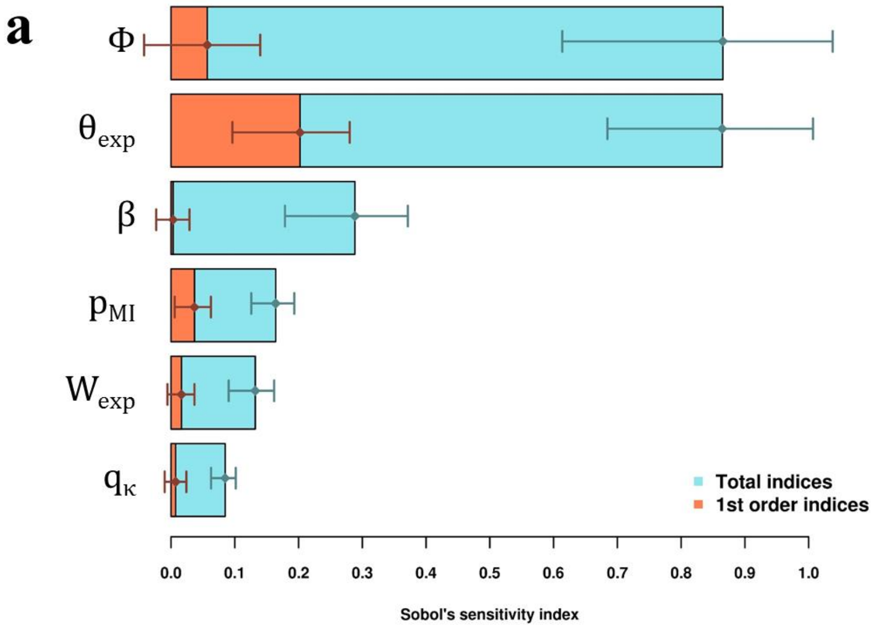

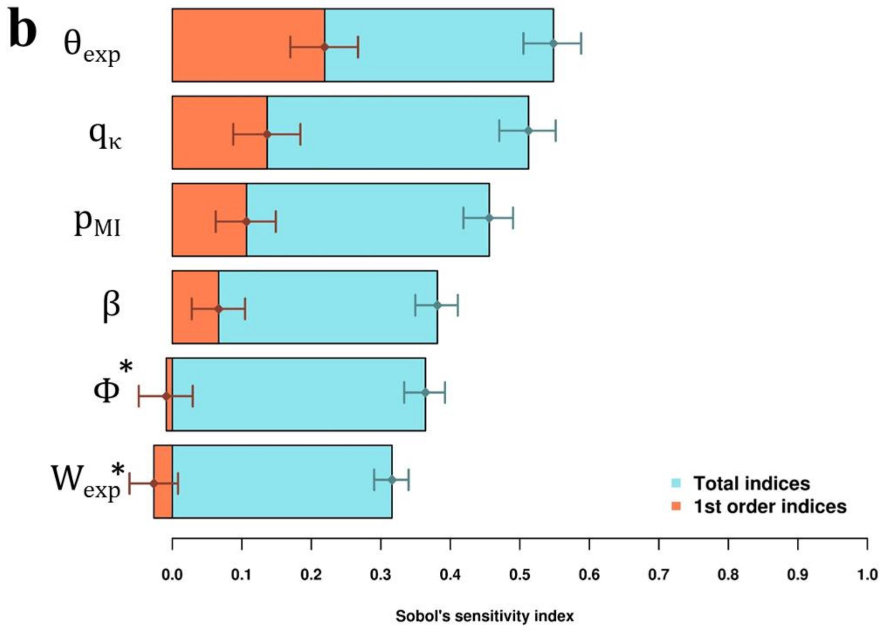

Figure S6. Sensitivity indices of the target parameters and their interactions up to the order 2 on the standard deviation of the output (σ_Y, standard deviation of the number of fully productive trees per hectare and per year) of the stochastic replicates, assessed through a degree-3 polynomial optimised by BIC in the broad-range analysis (a, R^2=0.48), or informed by knowledge acquired on sharka in peach orchards (b, R^2=0.62). θ_{exp}: expected duration of the latent period; Φ: probability of introduction at orchard planting; β: transmission coefficient; p_{MI}: relative probability of massive introduction; W_{exp}: expected value of the dispersal weighting variable; q_κ: quantile of the outgoing connectivity of the patch of first introduction.

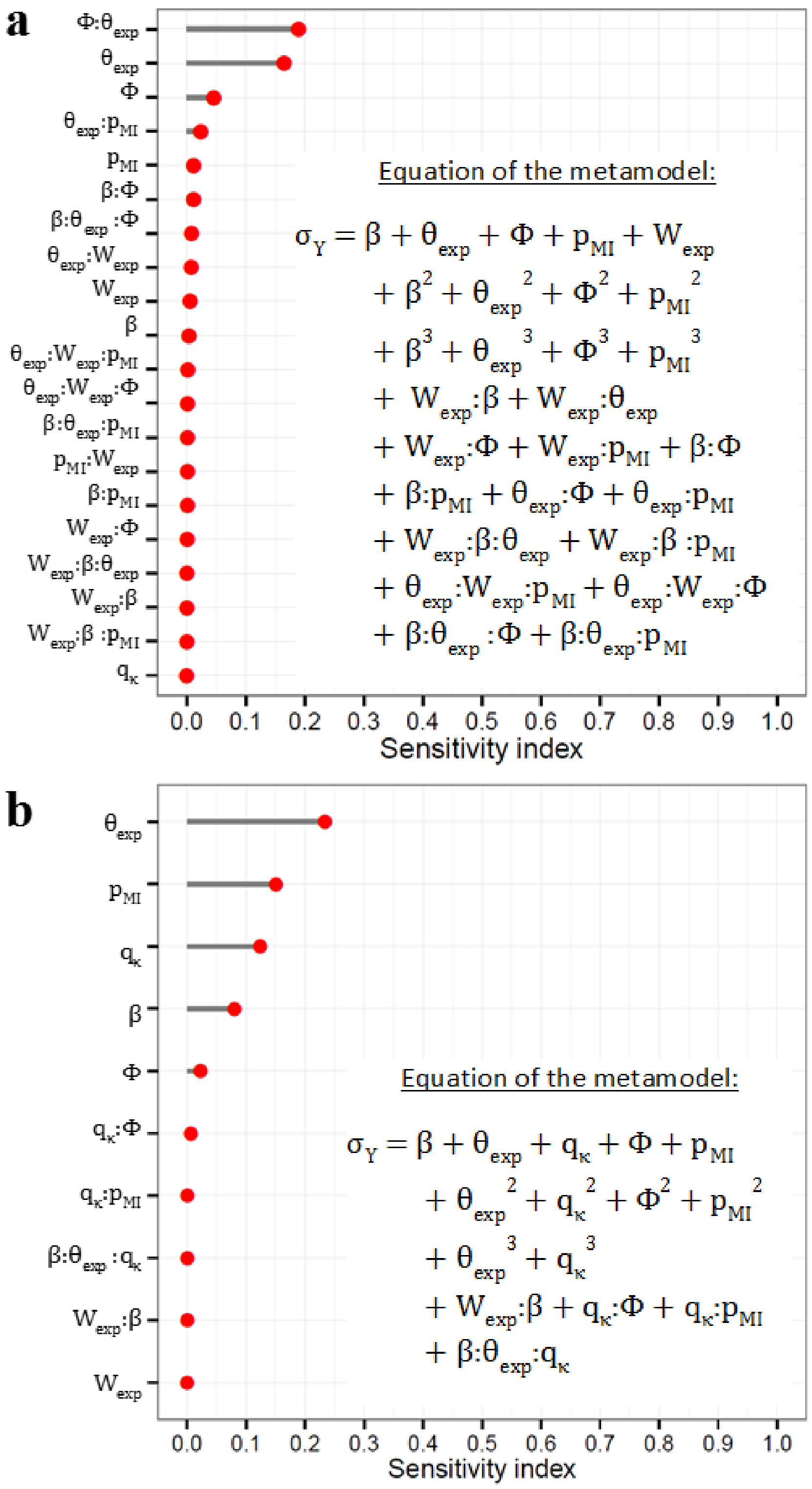

Figure S7. Effect of each target parameter on the standard deviation of the output (σ_Y, standard deviation of the equivalent number of fully productive trees per hectare and per year) in the broad-range analysis (a), or informed by knowledge acquired on sharka in peach orchards (b). Each point represents the standard deviation of 50 stochastic replicates of model simulations performed with the same combination of parameters. Blue line: prediction using the degree-3 polynomial optimised by BIC (all other parameters fixed at their mean value); this line is extremely close to the moving average of the number of productive trees, which is not represented for legibility. Green dashed line: standard deviation of the number of productive trees in the disease-free scenario. In (a) the thick line on the x-axis corresponds to the variation range used in the sharka-specific analysis; in (b) the full circle on the x-axis indicates the reference value of each parameter. W_{exp}: expected value of the dispersal weighting variable (higher values correspond to longer dispersal distances); β: transmission coefficient; θ_{exp}: expected duration of the latent period; q_κ: quantile of the outgoing connectivity of the patch of first introduction; Φ: probability of introduction at orchard planting; p_{MI}: relative probability of massive introduction.

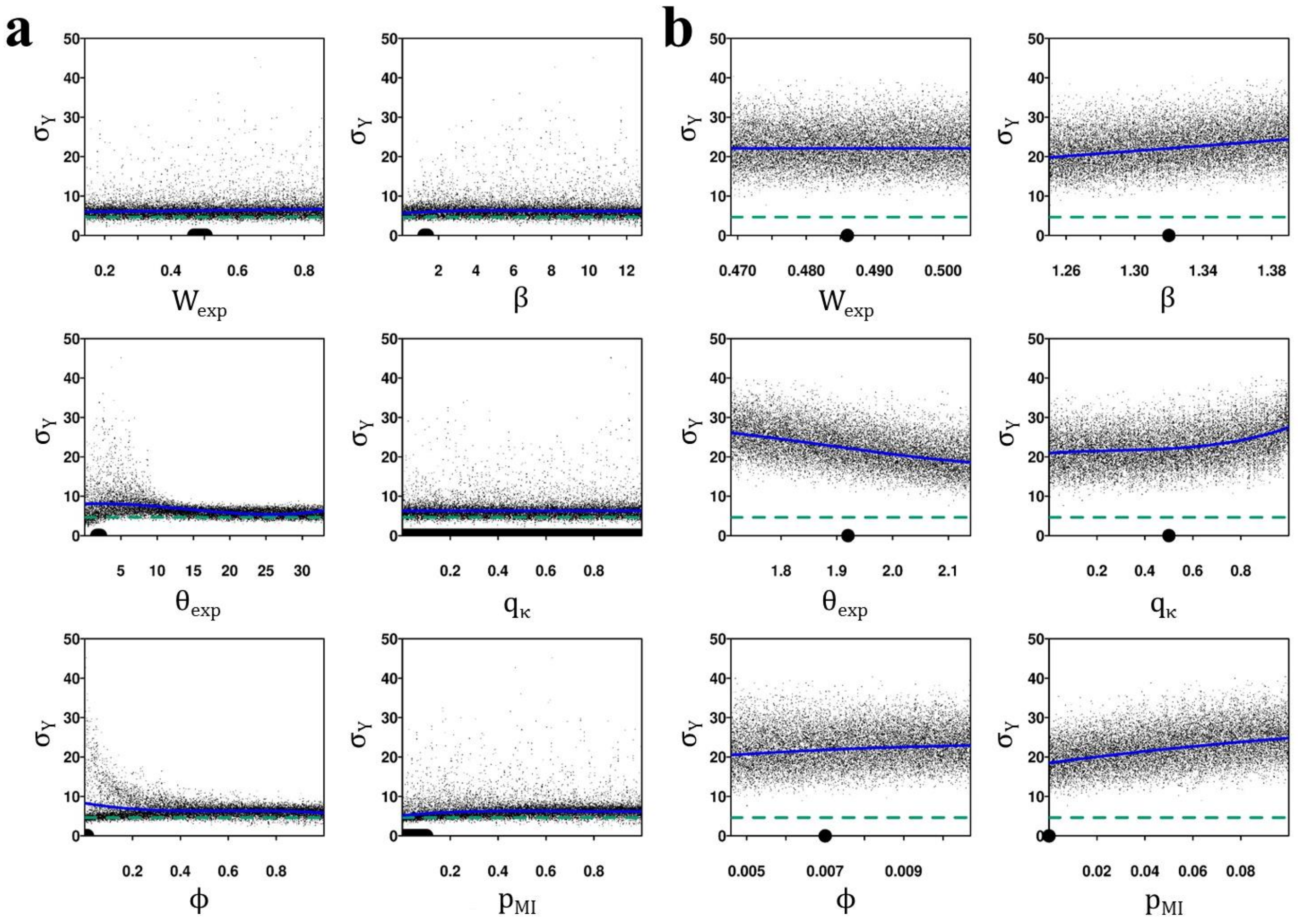

Figure S8. Robustness of the broad-range sensitivity analysis to changes in the upper bound of the expected duration of the latent period (from 5 to 33 years). The curves indicate 1st-order (solid lines) and total (dashed lines) Sobol's sensitivity indices of the 6 target parameters with regard to the mean output (μ_Y, mean number of fully productive trees per hectare and per year) of the stochastic replicates. The estimated 1st-order index may be higher than the estimated total index when the two indices are close and the number of simulations used to compute the indices is low.

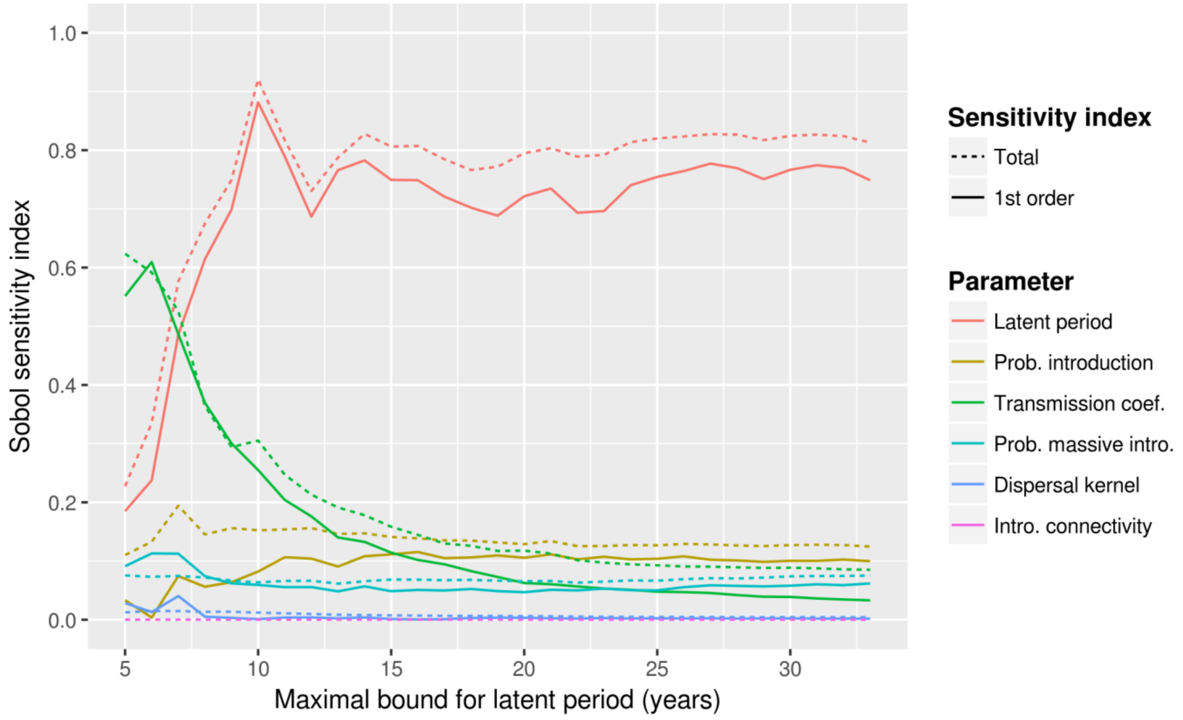

Figure S9. Sharka-specific sensitivity analysis on the mean output (μ_Y, mean of the equivalent number of fully productive trees per hectare and per year) but performed with a wide variation range (as in the broad-range analysis) for W_{exp}. a: 1st-order and total Sobol's sensitivity indices of the 6 target parameters ($\sum SI_1 = 0.88$). Horizontal bars: CI_{95} (assessed with 100 bootstrap replicates). b: Effect of each target parameter. Red line: polynomial regression; green dashed line: number of productive trees in the disease-free scenario; full circle on the x-axis: reference value of each parameter. W_{exp}: expected value of the dispersal weighting variable; θ_{exp}: expected duration of the latent period; Φ: probability of introduction at orchard planting; β: transmission coefficient; p_{MI}: relative probability of massive introduction; q_K: quantile of the outgoing connectivity of the patch of first introduction.

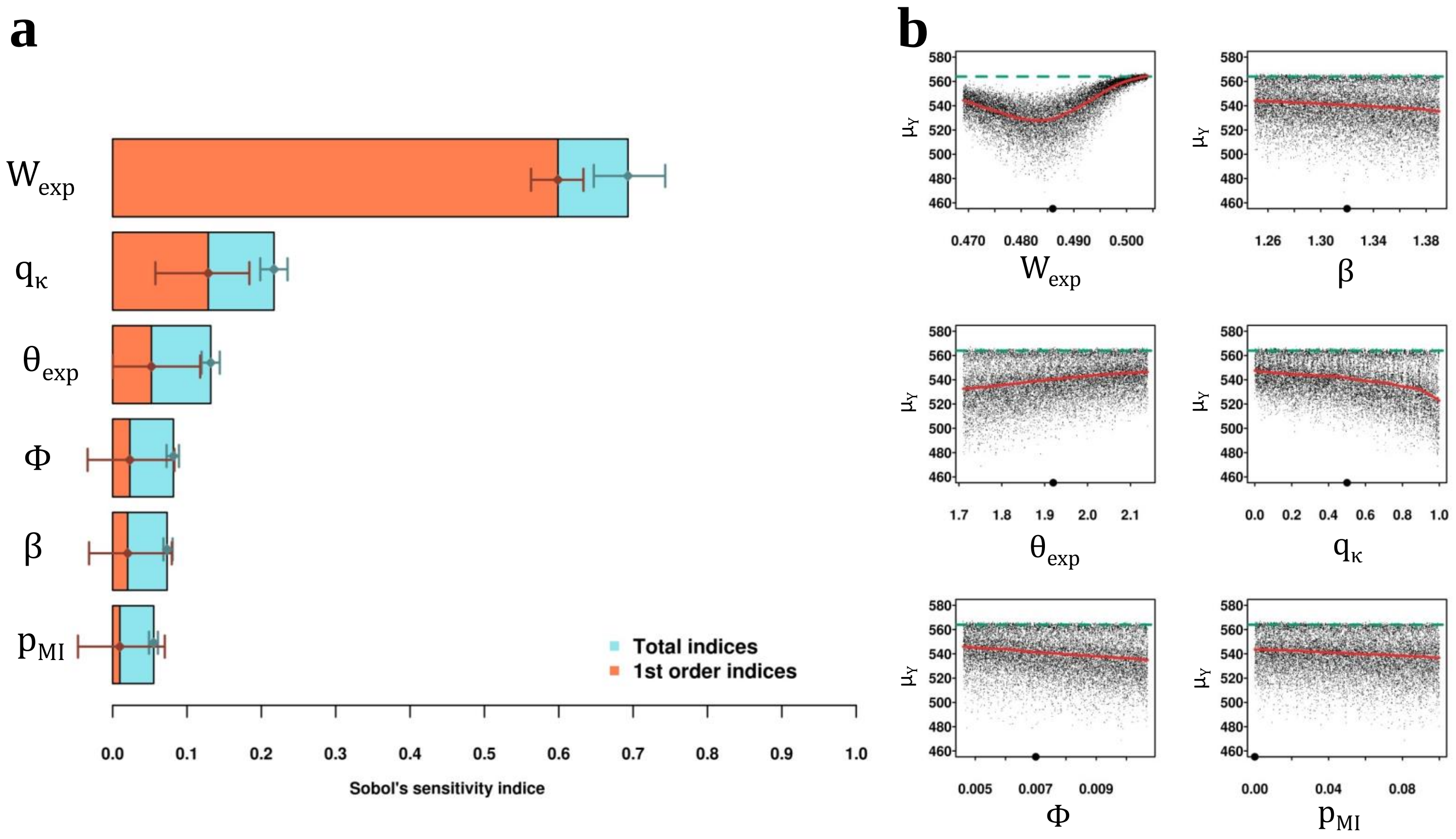

Methods S1. Relation between the mean and standard deviation of the latent period duration

In order to assess the relation between the mean and standard deviation of the latent period duration, we first studied the analogous relation for the incubation period, for which more data are available. Many studies dealing with the incubation period of infectious diseases suggest a correlation between the mean or median duration of the incubation period and its variance. In order to investigate this hypothesis, we estimated a proportionality coefficient between the mean (μ) and standard deviation (σ) of the incubation period duration for several human, animal, and plant infections.

When the duration of the incubation period was described by a lognormal distribution [1-3], we used the following approach:

> Let X be the duration of the incubation period and $Y = ln(X)$. $X \sim Lognormal(\mu_Y, \sigma_Y^2)$ implies $Y \sim Normal(\mu_Y, \sigma_Y^2)$; $E(Y) = \mu_Y$; and $V(Y) = \sigma_Y^2$. We note: $E(X) = \mu$, $M(X) = e^{\mu_Y}$, and $V(X) = \sigma^2$ the mean, median, and standard deviation of the incubation period duration, respectively.
>
> The concerned studies reported the median duration of the incubation period, $\boldsymbol{M(X)}$, and a dispersion factor, noted ***f***, and corresponding to $f = e^{\sigma_Y}$ here. It was then possible to estimate $\boldsymbol{\mu}$ and $\boldsymbol{\sigma}$ from these elements:
>
> $$\mu = e^{\mu_Y + \frac{\sigma_Y^2}{2}} = M(X) \times e^{\left(\frac{ln(f)^2}{2}\right)}$$
>
> $$\sigma^2 = e^{2\times\mu_Y + \sigma_Y^2} \times \left(e^{\sigma_Y^2} - 1\right) = M(X)^2 \times e^{(ln(f)^2)} \times \left(e^{(ln(f)^2)} - 1\right).$$

When only the 2.5% and 97.5% quantiles of the distribution of incubation durations (noted $q_{0.025}^X = e^{\mu_Y - \sigma_Y \times q_{0.975}^Y} = 14$ and $q_{0.975}^X = e^{\mu_Y + \sigma_Y \times q_{0.975}^Y} = 90$, respectively) were available [4], we estimated μ_Y and σ_Y using:

> $$\mu_Y = \frac{ln(q_{0.975}^X) + ln(q_{0.025}^X)}{2}$$
>
> $$\sigma_Y = \frac{ln(q_{0.975}^X) - ln(q_{0.025}^X)}{2\times q_{0.975}^Y}.$$
>
> Then, $\boldsymbol{\mu}$ and $\boldsymbol{\sigma}$ were estimated using the equations previously presented**.**

When the incubation period was described by a gamma distribution [5, 6], the mean and standard deviation were calculated from the shape (α) and rate (β) parameters using: $\boldsymbol{\mu = \alpha/\beta}$ and $\boldsymbol{\sigma = \sqrt{\alpha/\beta^2}}$.

We then obtained proportionality coefficients from 0.12 to 0.86 between μ and σ (Table S1). Additionally, in a study focused on a plant fungus [6], age-dependent incubation periods were measured and showed a significant correlation between the mean and standard deviation of this period, with an estimate proportionality coefficient of 0.23 (Figure S8).

In our model, the latent period is considered synchronised with the incubation period. The proportionality coefficient of 0.35 estimated in [7] is within the range of the coefficients estimated for the incubation period of other infections. Therefore, in each simulation of our model, the standard deviation of the latent period duration is calculated by multiplying its mean by 0.35.

Figure S10. Relation between the mean (μ) and standard deviation (σ) of the incubation period duration in *Rhizoctonia solani*. Data: [6]. Inset: relation for mean incubation periods between 0 and 2, which are better spread along the x-axis. Black lines: linear regressions.

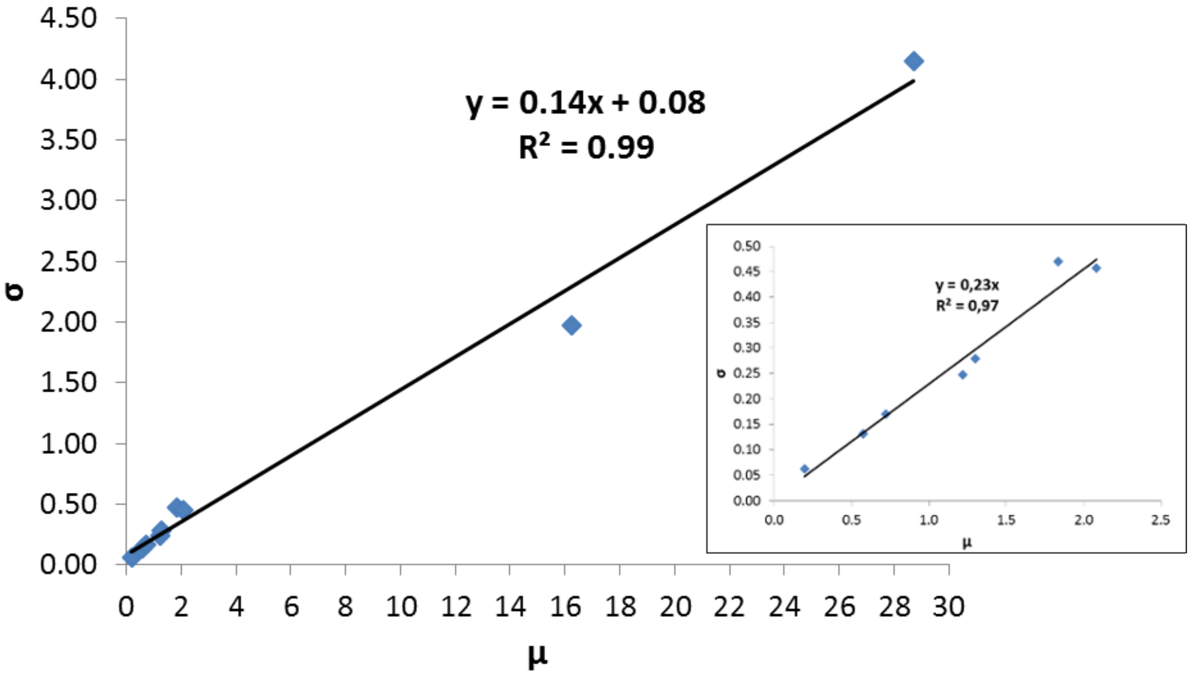

Table S1. Proportionality coefficient between the mean (μ) and standard deviation (σ) of the incubation or latent period durations in several human, animal, and plant infections.

Pathosystem	Lognormal distribution		Gamma distribution		Moments		Coef.	Reference
	M	f	α	β	μ	σ	σ/μ	
**Bacterial food poisoning**	0.1	1.48	-	-	0.10	0.04	0.41	[3]
**Streptococcal sore throat**	2.3	1.53	-	-	2.55	1.14	0.45	
Salmonella	2.4	1.47	-	-	2.58	1.03	0.40	
Salmonella enterica	2.4	1.51	-	-	2.61	1.12	0.43	
Poliovirus	11.9	1.50	-	-	12.92	5.46	0.42	
Measles virus	12.2	1.18	-	-	12.37	2.06	0.17	
Measles virus	12.4	1.32	-	-	12.89	3.65	0.28	
Measles virus	11.6	1.32	-	-	12.06	3.41	0.28	
Salmonella typhi	8.9	1.42	-	-	9.46	3.42	0.36	
Salmonella typhi	13.3	1.58	-	-	14.77	7.12	0.48	
Varicella zoster virus	14	1.14	-	-	14.12	1.86	0.13	
Varicella zoster virus	15.0	1.15	-	-	15.15	2.13	0.14	
Entamoeba histolytica	21.4	2.11	-	-	28.28	24.43	0.86	
Hepatitis B virus	100.0	1.24	-	-	102.34	22.27	0.22	
Hepatitis B virus	105.0	1.27	-	-	108.04	26.20	0.24	
**Monkey poliomyelitis**	8.6	1.31	-	-	8.92	2.45	0.28	
**Common cold**	2.4	1.50	-	-	2.61	1.10	0.42	
Plasmodium	6.4	1.33	-	-	6.67	1.94	0.29	
Adenovirus	5.6	1.26	-	-	5.75	1.35	0.23	[2]
Human coronavirus	3.2	1.15	-	-	3.23	0.45	0.14	
SARS-associated coronavirus	4.0	1.81	-	-	4.77	3.10	0.65	
Influenza A virus	1.4	1.51	-	-	1.52	0.66	0.43	
Influenza B virus	0.6	1.51	-	-	0.65	0.28	0.43	
Measles virus	12.5	1.23	-	-	12.77	2.67	0.21	
Parainfluenza virus	2.6	1.35	-	-	2.72	0.83	0.31	
Respiratory syncytial virus	4.4	1.24	-	-	4.50	0.98	0.22	
Rhinovirus	1.9	1.68	-	-	2.17	1.21	0.56	
Astrovirus	4.5	1.22	-	-	4.59	0.92	0.20	[1]
Norovirus	1.2	1.64	-	-	1.36	0.71	0.53	
***Norovirus* - Genogroup I**	1.1	1.82	-	-	1.32	0.86	0.66	
***Norovirus* - Genogroup II**	1.2	1.56	-	-	1.32	0.62	0.47	
Sapovirus	1.7	1.48	-	-	1.84	0.75	0.41	
Rotavirus	2.0	1.37	-	-	2.10	0.68	0.32	
Rabies virus	-	-	-	-	39.73	19.97	0.50	[4]
Yellow fever virus	-	-	1.8	8.1	14.58	10.87	0.75	[5]
Rhizoctonia solani	-	-	9.83	0.02	0.20	0.06	0.32	[6]
	-	-	19.19	0.03	0.58	0.13	0.23	
	-	-	18.00	0.04	0.72	0.17	0.24	
	-	-	24.39	0.05	1.22	0.25	0.20	
	-	-	21.68	0.06	1.30	0.28	0.21	
	-	-	15.30	0.12	1.84	0.47	0.26	
	-	-	20.84	0.10	2.08	0.46	0.22	
	-	-	67.72	0.24	16.25	1.98	0.12	
	-	-	47.88	0.60	28.73	4.15	0.14	
Plum pox virus	**-**	**-**	**-**	**-**	**1.92**	**0.66**	**0.35**	**[7]**

References

1 Lee, R., Lessler, J., Lee, R., Rudolph, K., Reich, N., Perl, T., Cummings, D. 2013 Incubation periods of viral gastroenteritis: a systematic review. *BMC Infect. Dis.* **13**, 446. (doi:10.1186/1471-2334-13-446)

2 Lessler, J., Reich, N. G., Brookmeyer, R., Perl, T. M., Nelson, K. E., Cummings, D. A. T. 2009 Incubation periods of acute respiratory viral infections: a systematic review. *Lancet Infect. Dis.* **9**, 291-300. (doi:10.1016/S1473-3099(09)70069-6)

3 Sartwell, P. E. 1950 The distribution of incubation periods of infectious disease. *Am. J. Hyg.* **51**, 310-318.

4 Deal, B., Farello, C., Lancaster, M., Kompare, T., Hannon, B. 2000 A dynamic model of the spatial spread of an infectious disease: the case of fox rabies in Illinois. *Environ. Model. Assess.* **5**, 47-62. (doi:10.1023/A:1019045224429)

5 Johansson, M. A., Arana-Vizcarrondo, N., Biggerstaff, B. J., Staples, J. E. 2010 Incubation periods of Yellow fever virus. *Am. J. Trop. Med. Hyg.* **83**, 183-188. (doi:10.4269/ajtmh.2010.09-0782)

6 Leclerc, M., Doré, T., Gilligan, C. A., Lucas, P., Filipe, J. A. N. 2014 Estimating the delay between host infection and disease (incubation period) and assessing its significance to the epidemiology of plant diseases. *PLoS ONE*. **9**, e86568. (doi:10.1371/journal.pone.0086568)

7 Pleydell, D. R. J., Soubeyrand, S., Dallot, S., Labonne, G., Chadœuf, J., Jacquot, E., Thébaud, G. 2017 Estimation of the dispersal distances of an aphid-borne virus in a patchy landscape. *bioRxiv*. (doi:10.1101/109561)

Methods S2. Bounds for the expected value of the latent period duration and the transmission coefficient in the broad-range sensitivity analysis

The broad-range sensitivity analysis (i.e. the analysis designed to assess the intrinsic influence of the target parameter on model behaviour), required to define appropriate minimal and maximal bounds of the variation ranges of parameters whose definition domain is not finite. Thus, we tried to find values below and above which the epidemic process does not fundamentally change any more. For example, the mean duration of the latent period (θ_{exp}) can be left-bounded by 1 day. Indeed, lower values would result in similar processes, since the time step of the model is one week. The estimate of the maximal bound is less easy. We assumed that the epidemic process is unchanged for θ_{exp} values above θ_{exp}^{max}, for which only 5% of the infected hosts at orchard planting become infectious before orchard removal. This value was calculated as follows:

Let Ψ describe the duration of orchard cultivation; $\Psi \sim \text{Poisson}(\psi = 15)$.

Let Θ describe the duration of the latent period; $\Theta \sim Gamma(\theta_{exp}, 0.35 \times \theta_{exp})$ (parameterising the Gamma distribution with its expectation and variance; see also Methods S1).

We seek θ_{exp}^{max} such that:

$P(\Theta \leq \Psi) = \sum_{x=0}^{+\infty}\{P(\Psi = x) \times P(\Theta \leq x)\} = 5\%$.

$P(\Theta \leq \Psi)$ equals 5.43% with θ_{exp}=32; 4.77% with θ_{exp}=33; and 4.19% with θ_{exp}=34. Thus θ_{exp}^{max} was estimated at 33.

The results of the broad-range sensitivity analysis validated our hypothesis because the model output was stable for θ_{exp} values beyond 15 years (Figure 4a), and *a fortiori* beyond 33 years.

The minimal and maximal bounds for β (transmission coefficient) were assessed using a deterministic susceptible-infectious model in a single orchard (in the patch of the landscape with median area, noted $A_{0.5}$=0.81 ha and indexed by $i_{0.5}$) where only 1 host was infected at planting. Let S_t and I_t be the number of susceptible and infectious trees, respectively. The total number of trees in this orchard is $S_t + I_t = A_{0.5} \times \mu^{th} = N_0$ (with μ^{th}=666 trees/ha the reference density planting estimated through expert opinion). After n years of epidemic, the number of infectious trees, I_T ($T = n \times 52$ since the time step is one week), can be estimated using the following algorithm:

Initial conditions: $P(S_0) = \frac{N_0 - 1}{N_0}$ and $I_0 = 1$;

Then for each time step t (t=0,...,T-1):

- $\lambda_t = \frac{\beta \times \alpha_t \times m_{i_{0.5}, i_{0.5}}}{N_0} \times I_t$
- $P(S_{t+1}) = P(S_t) \times e^{-\lambda_t}$
- $I_{t+1} = N_0 \times [1 - P(S_{t+1})]$

The minimal bound (β=0.08) corresponds to the value for which $I_t = 2$ after n=30 years, and the maximal bound (β=12.80) corresponds to the value for which $I_t = 0.95 \times N_0$ after n=3 years (i.e. 95% of the orchard is infectious when the orchard just becomes productive; in such extreme scenario, prunus cultivation would be economically unfeasible). The broad-range sensitivity analysis confirmed that the epidemic process does not change anymore beyond this maximal value, as shown by the stabilisation of the output variable for β values beyond 5.00 (Figure 4a).

Methods S3. Computation of Sobol's sensitivity indices

Let $Y = f(X_1, \dots, X_p)$ be a function of a set of input parameters X_i (i=1,…,p) defined on a p-dimensional cube. Assuming that these input parameters are independent, the function can be decomposed into uncorrelated terms of increasing dimensionality [1-3], using conditional expectations of Y given X_i:

$$Y = f(X_1, \dots, X_p) = f_0 + \sum_{i=1}^{p} f_i(X_i) + \sum_{1 \le i < j \le p} f_{i,j}(X_i, X_j) + \sum_{1 \le i < j < k \le p} f_{i,j,k}(X_i, X_j, X_k) + \cdots + f_{1,2,\dots,p}(X_1, \dots, X_p)$$

where

$f_0 = E(Y)$;
$f_i = E(Y|X_i) - f_0$;
$f_{i,j} = E(Y|X_i, X_j) - E(Y|X_i) - E(Y|X_j) + f_0$;
$f_{i,j,k} = E(Y|X_i, X_j, X_k) - E(Y|X_i, X_j) - E(Y|X_i, X_k) - E(Y|X_j, X_k) + E(Y|X_i) + E(Y|X_j) + E(Y|X_k) - f_0$.

Moreover, the variance of Y can be decomposed as a sum of the variances associated with the different terms:

$$V(Y) = \sum_{i=1}^{p} V(f_i) + \sum_{1 \le i < j \le p} V(f_{i,j}) + \sum_{1 \le i < j < k \le p} V(f_{i,j,k}) + \cdots + V(f_{1,2,\dots,p}).$$

Then the main contribution, or main effect, of X_i to $V(Y)$ is quantified by the 1st-order index $SI_1^{X_i}$:

$$SI_1^{X_i} = \frac{V(f_i)}{V(Y)} = \frac{V[E(Y|X_i)]}{V(Y)}.$$

This index quantifies the contribution of the regression $E(Y|X_i)$ to the variance of Y. The total effect of X_i including all its interactions with other parameters is quantified by the total sensitivity index $SI_{tot}^{X_i}$:

$$SI_{tot}^{X_i} = \frac{V(f_i) + \sum_{j=1}^{p} V(f_{i,j}) + \sum_{1 \le j < k \le p} V(f_{i,j,k}) + \cdots + V(f_{1,2,\dots,p})}{V(Y)}$$

which is equal to:

$$SI_{tot}^{X_i} = \frac{E[V(Y|X_{-i})]}{V(Y)}$$

where X_{-i} denotes the whole set of parameters except X_i. $SI_1^{X_i}$ and $SI_{tot}^{X_i}$ are defined in [0,1], and a parameter is considered noninfluential if and only if $SI_{tot}^{X_i}$=0.

References

1 Saltelli, A., Chan, K., Scott, E. M. 2000 *Sensitivity Analysis*. London: Wiley.

2 Saltelli, A., Ratto, M., Andres, T., Campolongo, F., Cariboni, J., Gatelli, D., Saisana, M., Tarantola, S. 2008 *Global Sensitivity Analysis, The Primer*. Wiley.

3 Sobol, I. M. 1993 Sensitivity analysis for non-linear mathematical models. *Math. Modell. Comput. Exp.* **1**, 407-414.

Videos. Simulations of different scenarios of sharka epidemics in a French peach production area in the absence of disease control (one year every 0.5 second). Each polygon represents a patch on which orchards are cultivated, with a turnover of 1/15 $year^{-1}$. Orchard colour represents the proportion of healthy trees, from 100% (white, all trees in the S state) to 0% (red, all trees in the E or I states). The green area is not cultivated with prunus. Except the introduction parameters, all model parameters were fixed at their reference value. ‘Single introduction’ means only one pathogen introduction at year 1, whereas ‘multiple introductions’ means a first introduction at year 1 and a risk of subsequent introductions at every orchard planting.

Video S1. scenario B) Single PPV introduction through 1 infectious tree in a moderately connected patch.

Video S2. scenario C) Single PPV introduction through several infectious trees in a moderately connected patch.

Video S3. scenario D) Multiple PPV introductions through several infectious trees, with 1st introduction in a weakly connected patch.

Video S4. scenario E) Multiple PPV introductions through several infectious trees, with 1st introduction in a moderately connected patch (reference scenario).

Video S5. scenario F) Multiple PPV introductions through several infectious trees, with 1st introduction in a highly connected patch.
